# Enhancing Tumor Immunotherapy by Multivalent Anti‐PD‐L1 Nanobody Assembled via Ferritin Nanocage

**DOI:** 10.1002/advs.202308248

**Published:** 2024-03-16

**Authors:** Manman Liu, Duo Jin, Wenxin Yu, Jiaji Yu, Kaiming Cao, Junjie Cheng, Xiaohu Zheng, Andrew Wang, Yangzhong Liu

**Affiliations:** ^1^ Department of Pharmacy the First Affiliated Hospital of USTC Division of Life Sciences and Medicine Department of Chemistry University of Science and Technology of China Hefei 230001 China; ^2^ The CAS Key Laboratory of Innate Immunity and Chronic Disease School of Basic Medical Sciences Center for Advanced Interdisciplinary Science and Biomedicine of IHM Division of Life Sciences and Medicine University of Science and Technology of China Hefei 230027 China; ^3^ Department of Radiation Oncology University of Texas Southwestern Medical Center Dallas 75230 USA

**Keywords:** ferritin, immunotherapy, multivalent nanobody, PD‐L1

## Abstract

Increasing immunotherapy response rate and durability can lead to significant improvements in cancer care. To address this challenge, a novel multivalent immune checkpoint therapeutic platform is constructed through site‐specific ligation of anti‐PD‐L1 nanobody (Nb) on ferritin (Ftn) nanocage. Nb‐Ftn blocks PD‐1/PD‐L1 interaction and downregulates PD‐L1 levels via endocytosis‐induced degradation. In addition, the cage structure of Ftn allows encapsulation of indocyanine green (ICG), an FDA‐approved dye. Photothermal treatment with Nb‐Ftn@ICG induces immunogenic death of tumor cells, which improves systemic immune response via maturation of dendritic cells and enhanced infiltration of T cells. Moreover, Nb‐Ftn encapsulation significantly enhances cellular uptake, tumor accumulation and retention of ICG. In vivo assays showed that this nanoplatform ablates the primary tumor, suppresses abscopal tumors and inhibits tumor metastasis, leading to a prolonged survival rate. This work presents a novel strategy for improving cancer immunotherapy using multivalent nanobody‐ferritin conjugates as immunological targeting and enhancing carriers.

## Graphical abstract

A multivalent PD‐L1 nanobody (Nb) is constructed via assembly of ferritin nanocage (Ftn). With encapsulation of photosensitizer and photothermal therapy, the Nb‐Ftn conjugate significantly enhances the immunotherapeutic response via DC maturation and enhanced T‐cell infiltration, resulting in efficient ablation of primary tumor, inhibition of distal tumor and suppression of metastasis, leading to a prolonged survival rate.

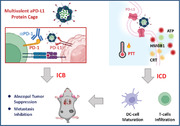

## 1 Introduction

Cancer immunotherapy is a remarkable approach in cancer treatment that stimulates the immune system to eliminate malignant cells and achieves a durable clinical response.^[^ ^1^ ^]^ Over the past decade, the discovery of monoclonal antibody (mAb)‐based immune checkpoint blockade (ICB) has substantially improved cancer immunotherapy.^[^ ^2^ ^]^ In particular, mAbs against PD‐1/PD‐L1 have drawn great attention due to their successful application in the clinic for treating a variety of cancers.^[^ ^3^ ^]^ Nevertheless, only a limited proportion of patients (20–40%) benefit from this treatment.^[^ ^4^ ^]^ Insufficient infiltration of T cells to “cold cancer” is typically associated with a low immune response to ICB therapy.^[^ ^5^ ^]^ To address this issue, an adaptive immune response strategy has been applied by inducing immunogenic cell death (ICD), which causes the release of tumor‐associated antigens (TAAs) and damage‐associated molecular patterns (DAMPs).^[^ ^6^ ^]^ This process promotes the maturation of dendritic cells (DCs) and stimulates the activation and infiltration of tumor‐specific T cells. Among various ICD strategies, photothermal therapy has emerged as a promising approach for evoking an immune response by transforming immunological “cold tumors” into “hot tumors”.^[^ ^7^ ^]^ It has been found that mild photothermal therapy (PTT) increases immunotherapy of PD‐L1 mAbs by increasing the recruitment of tumor‐infiltrating lymphocytes and boosting T‐cell activity against tumors.^[^ ^7c^ ^]^

Increasing binding valency is an effective strategy to improve the functional affinity, decrease dissociation rates, and enhance pharmacokinetics and biodistribution of antibodies; hence, recombinant multimeric antibodies have shown great advantages over conventional intact antibodies.^[^ ^8^ ^]^ However, engineering multivalent mAbs is a challenge due to the large size and precise structure of the molecules. Nanobody is the smallest naturally occurring antibody possessing antigenic affinity and specificity similar to those of conventional antibodies.^[^ ^9^ ^]^ Compared with conventional antibody, nanobody exhibits the advantages of easy modification, tumor penetration, low immunogenicity and easy large‐scale synthesis.^[^ ^10^ ^]^ Nevertheless, nanobody often suffers from rapid renal clearance due to its small size. Multivalent nanobodies, including nanobody‐ferritin fusion, demonstrate prolonged in vivo circulation, enhanced affinity and excellent physical stability.^[^ ^11^ ^]^ Ferritin is a cage‐like protein that can be used for delivering a wide range of therapeutic cargos.^[^ ^12^ ^]^ We have previously shown that the nanobody‐ferritin platform can be constructed using a post‐protein conjugation strategy without interfering with the properties of either component.^[^ ^13^ ^]^ We proposed that the nanobody‐ferritin (Nb‐Ftn) nanoplatform can be applied for synergistic enhancement of immunotherapy through rational design of the construction elements.

In this work, we constructed an Nb‐Ftn nanoplatform using an anti‐PD‐L1 nanobody for cancer immunotherapy. Anti‐PD‐L1 nanobody was site‐specifically conjugated to ferritin to target PD‐L1‐overexpressing tumor cells. Indocyanine green (ICG), an FDA‐approved photothermal agent,^[^ ^14^ ^]^ was encapsulated in the cavity of ferritin for PTT. This nanobody‐ferritin nanoplatform (Nb‐Ftn@ICG) demonstrated several benefits, including targeted accumulation and retention in tumor tissues, induction of immunogenic death of tumor cells following laser irradiation, maturation of dendritic cells and T‐cell immune response, and immune checkpoint blockade and degradation of PD‐L1. The nanobody‐ferritin nanoplatform, by integrating the efficacy of PTT and immunotherapy, effectively ablated primary treated tumors, inhibited the growth of untreated tumors, and reduced metastasis of lung tumors, demonstrating the potential of this approach for cancer therapy.

## 2 Results and Discussion

### 2.1 Preparation and Characterization of Nb‐Ftn@ICG

**Scheme 1:**
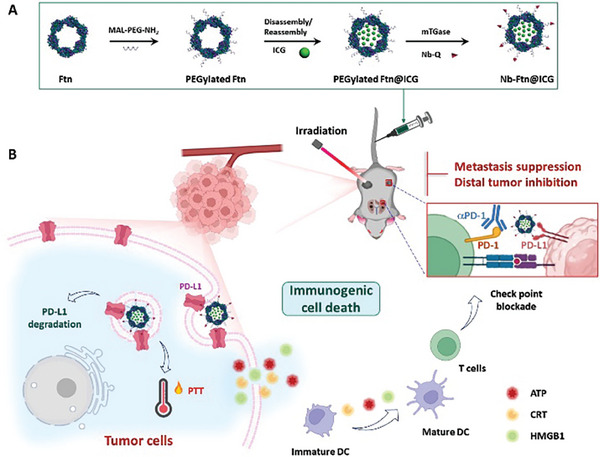
Schematic illustration of the preparation of Nb‐Ftn@ICG and its functions in immunotherapy.

**Figure 1:**
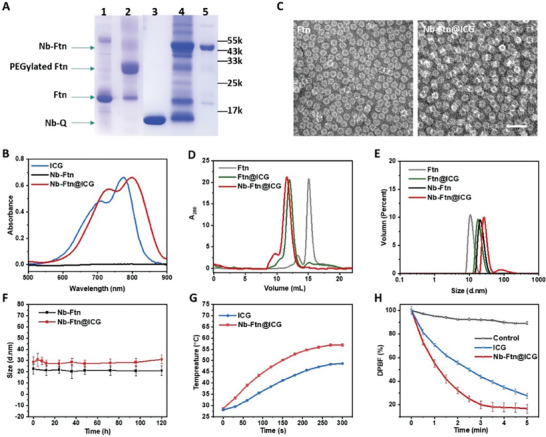
Preparation and characterization of Nb‐Ftn@ICG. A) SDS‐PAGE analysis of the synthesis of Nb‐Ftn. Lane 1: Ftn; lane 2: production of PEGylated Ftn; lane 3: Nb‐Q; lane 4: products of the conjugation between PEGylated Ftn and Nb‐Q catalyzed by mTGase; lane 5: purified Nb‐Ftn. B) UV–vis spectra of ICG and Nb‐Ftn@ICG in PBS buffer. C) TEM photograph of Ftn and Nb‐Ftn@ICG. Scale bar = 40 nm. D) SEC analysis of Ftn, PEGylated Ftn@ICG and Nb‐Ftn@ICG. E) Dynamic light scattering (DLS) analysis of Ftn, PEGylated Ftn@ICG, Nb‐Ftn and Nb‐Ftn@ICG. F) Stability assessment of Nb‐Ftn and Nb‐Ftn@ICG by measuring hydrodynamic diameter in RPMI‐1640 for 120 h. Data are shown as the mean ± standard deviation (SD, n = 3). G) Temperature changes of ICG (20 µg mL^−1^) and Nb‐Ftn@ICG solution (equivalent to 20 µg mL^−1^ free ICG) under 808 nm laser irradiation (1 W cm^−2^) for 5 min. H) ROS generation of ICG (5 µg mL^−1^) and Nb‐Ftn@ICG (equivalent to 5 µg mL^−1^ free ICG) under 808 nm laser irradiation (1 W cm^−2^) using DPBF as a probe. DPBF solution was used as a control. Data are shown as the mean ± standard deviation (SD, n = 3).

The preparation of the Nb‐Ftn@ICG nanoplatform is schematically illustrated in **Scheme** **1**. A maleimido and amino bifunctionalized PEG (MAL‐PEG‐NH_2_) was used to link Nb and Ftn. First, the Ftn nanocage was site‐specifically PEGylated via a click reaction between the maleimide of PEG and the cysteine residues on the surface of Ftn. Gel electrophoresis analysis showed that over 90% of the 24‐mer Ftn was PEGylated (lane 2 in **Figure** **1**A). Next, ICG was loaded into the PEGylated Ftn through a disassembly/reassembly process to form Ftn@ICG. ≈100 ICG molecules were encapsulated into each Ftn cavity according to the measurement of the UV absorbance of ICG.

Anti‐PD‐L1 Nb was expressed with C‐terminal fusion of a LLQS tag (Nb‐Q) by genetic engineering, which allows site‐specific conjugation with the NH_2_ group of PEG through a mTGase‐mediated ligation.^[^ ^13^, ^15^ ^]^ Hence, the anti‐PD‐L1 Nb can be linked to the PEGylated Ftn or Ftn@ICG, generating a nanobody‐ferritin platform (Nb‐Ftn or Nb‐Ftn@ICG). The enzyme‐catalyzed reaction was completed in 1 h at 25 °C. Gel electrophoresis confirmed the production of Nb‐Ftn at 43 kDa (lane 4 in Figure 1A), and a high purity product was obtained by size exclusive chromatography (lane 5 in Figure 1A). Although the ferritin conjugation can be reached to over 90% of the subunits, the Nb‐Ftn with conjugation of ≈50% of the subunits was used by controlling the ratio of nanobody in the ligation (Figure S1, Supporting Information, lane 4), as full conjugation is unnecessary for targeting activity.^[^ ^16^ ^]^

The encapsulation of ICG in Nb‐Ftn was confirmed by UV–vis spectroscopy. Nb‐Ftn@ICG exhibited strong absorbance at 798 nm and a shoulder at 732 nm, which was similar to the spectrum of free ICG, while Nb‐Ftn showed no absorbance in the range of 500–900 nm (Figure 1B). The slight red shift of the ICG peak after Ftn encapsulation is probably due to the interaction between ICG and Ftn. TEM images showed the spherical structure of Nb‐Ftn@ICG with a slightly increased size relative to Ftn, suggesting that the encapsulation of ICG did not disturb the structure of ferritin (Figure 1C). The increased molecular weight of Nb‐Ftn@ICG was confirmed by SEC analysis (Figure 1D). Dynamic light scattering (DLS) analysis showed that the hydrodynamic diameters of Nb‐Ftn and Nb‐Ftn@ICG were 22.47 ± 4.29 and 28.62 ± 4.87 nm, respectively (Figure 1E). Moreover, the hydrodynamic diameters of Nb‐Ftn and Nb‐Ftn@ICG remained consistent during incubation in RPMI‐1640 at 37 °C for 5 days (Figure 1F), indicating that Nb‐Ftn and Nb‐Ftn@ICG possess considerable stability.

### 2.2 Evaluation of Photothermal Effect and ROS Generation

The photothermal effect of Nb‐Ftn@ICG was investigated by measuring the temperature increase upon irradiation of ICG or Nb‐Ftn@ICG with an 808 nm laser. After 5 min of irradiation (1 W cm^−2^), the temperature elevated by 9.6–33.5 °C, depending on the concentration of Nb‐Ftn@ICG (Figure 1G; Figure S2A, Supporting Information). In contrast, no conspicuous temperature elevation was observed in the buffer without Nb‐Ftn@ICG (Figure S2A, Supporting Information). The temperature elevation was also dependent on the laser power (Figure S2B, Supporting Information). Interestingly, Nb‐Ftn@ICG demonstrated a more pronounced photothermal effect than free ICG, since the wavelength of the maximum absorption of Nb‐Ftn@ICG (798 nm) is closer to the wavelength of laser irradiation (808 nm) than free ICG (778 nm) (Figure 1B).

The ROS generation of Nb‐Ftn@ICG was measured using a DPBF probe, as ROS can bleach the absorption of DPBF at 417 nm. Upon 808 nm laser irradiation, Nb‐Ftn@ICG exhibited an improved ROS generation rate relative to free ICG, as indicated by the more rapid DPBF bleaching (Figure 1H). This result is consistent with the photothermal effect of the two agents, probably because the absorption of Nb‐Ftn@ICG (798 nm) is closer to the laser wavelength (808 nm) than that of free ICG (778 nm).

### 2.3 Cellular Uptake, Subcellular Localization and Inhibitory Effect of Nb‐Ftn@ICG

**Figure 2:**
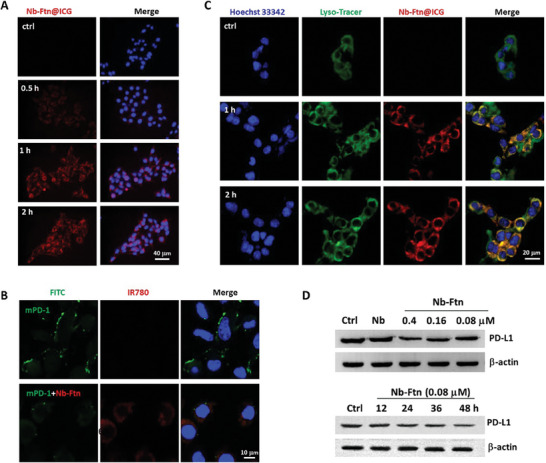
Cellular analyses of Nb‐Ftn@ICG. A) Cellular uptake of Nb‐Ftn@ICG measured via fluorescence imaging. Images were recorded after incubating the cells with Nb‐Ftn@ICG (0.6 µm) for 0.5, 1 and 2 h. Nb‐Ftn@ICG was shown in red, and nuclei were stained with Hoechst 33342 (blue). Scale bar = 40 µm. B) Confocal laser scanning microscopy (CLSM) images of cells after incubation with mPD‐1 (2 µm) or a mixture of mPD‐1 (2 µm) and Nb‐Ftn@ICG (0.2 µm) for 30 min. Nb‐Ftn@ICG was shown in red, mPD‐1 (a recombinant extracellular domain of the murine) was labeled with a green dye FITC, and nuclei were stained with Hoechst 33342. Scale bar = 10 µm. (C) CLSM images showing the lysosomal co‐localization of Nb‐Ftn@ICG. Images were recorded on cells incubated with Nb‐Ftn@ICG (0.6 µm) for 1 or 2 h. Nb‐Ftn@ICG was shown in red, lysosomes were stained with Lyso‐Green Tracker (green), and nuclei were stained with Hoechst 33342. Scale bar = 20 µm. D) Western blot analysis of the downregulatory effect of Nb‐Ftn on the cellular level of PD‐L1. Cells were treated with different concentrations of Nb‐Ftn for 24 h (upper portion) or 0.08 µm Nb‐Ftn for different time (lower portion).

PD‐L1‐positive B16F10 cells were chosen to analyze the cellular uptake of Nb‐Ftn@ICG. Fluorescence microscopy showed the time‐dependent uptake of Nb‐Ftn@ICG (**Figure** **2**A). By comparison, no detectable cellular uptake was observed after incubation with free ICG for 2 h (Figure S3, Supporting Information). This result confirmed that the Nb‐Ftn carrier facilitated the internalization of ICG, probably due to the presence of anti‐PD‐L1 Nb.

Next, we investigated whether Nb‐Ftn is capable of disrupting the interaction between PD‐1 and PD‐L1 using FITC‐labeled mPD‐1 (FITC‐mPD‐1). Confocal laser scanning microscopy (CLSM) imaging showed that mPD‐1 can bind to PD‐L1‐positive B16F10 cells, resulting in a noticeable green fluorescent signal on the cell membrane (Figure 2B); in comparison, the green fluorescence signal was diminished, and a red fluorescence signal of Nb‐Ftn@IR780 appeared after treating cells with a premix of Nb‐Ftn@IR780 and FITC‐mPD‐1, suggesting that Nb‐Ftn could block the PD‐1/PD‐L1 interaction on the cell surface. Moreover, a flow cytometry assay was performed to assess the blocking efficacy of Nb‐Ftn (Figure S4, Supporting Information). Similar to the CLSM images, cells treated with FITC‐mPD‐1 exhibited greater fluorescence intensity than cells incubated with a mixture of FITC‐mPD‐1 and Nb‐Ftn, while cells treated with Nb‐Ftn alone were used as a control. The decrease in fluorescence demonstrated the feasibility of using Nb‐Ftn as a PD‐1/PD‐L1 inhibitor.

The intercellular distribution of Nb‐Ftn@ICG was analyzed via fluorescence imaging. The result showed that the fluorescence of Nb‐Ftn@ICG (red) clearly overlapped with that of the lysosome tracer (green), resulting in a yellow color in the overlay images (Figure 2C). This result confirmed that a major portion of Nb‐Ftn@ICG was localized within lysosomes after cellular uptake, which is consistent with receptor‐binding‐mediated endocytosis.

### 2.4 Nb‐Ftn@ICG Degrades PD‐L1

As proteins can be degraded through a lysosomal pathway,^[^ ^17^ ^]^ we speculated that the lysosomal location of Nb‐Ftn@ICG can degrade PD‐L1 as it was internalized via PD‐L1 binding. Western blot assay clearly showed that the PD‐L1 in tumor cells was degraded after treatment with Nb‐Ftn in a time‐ and concentration‐dependent manner (Figure 2D). Quantification of the bands indicated that the PD‐L1 level decreased to 48% after incubation with 0.4 µm Nb‐Ftn for 24 h, and 0.08 µm Nb‐Ftn reduced the PD‐L1 level to 54% in 48 h (Figure S5, Supporting Information). By comparison, treatment with the unmodified anti‐PD‐L1 Nb (4 µm) had only a marginal effect on the PD‐L1 levels in cells. These results revealed that the Nb‐Ftn nanoplatform can target PD‐L1‐expressing tumor cells, interfere with the PD‐1/PD‐L1 interaction, and trigger lysosomal degradation of PD‐L1.

### 2.5 Cytotoxicity of Nb‐Ftn@ICG

**Figure 3:**
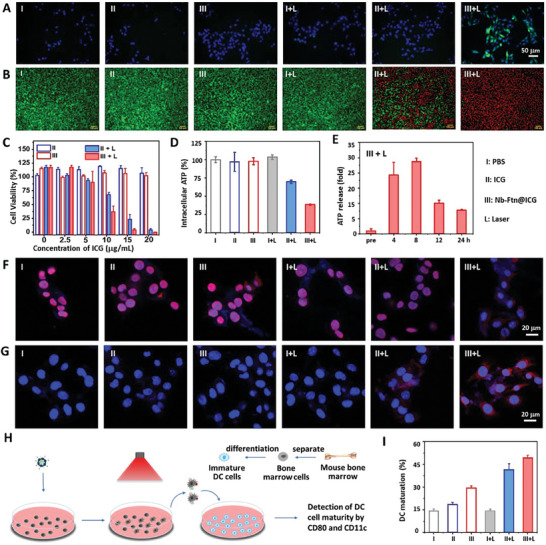
ROS generation, cytotoxicity, and ICD‐inducing effect of Nb‐Ftn@ICG upon PTT. A) Fluorescence imaging of ROS generation in cells using a ROS assay kit. Cells were treated with ICG or Nb‐Ftn@ICG at 37 °C for 2 h. After changing the medium to fresh medium, the cells were irradiated with an 808 nm laser (1 W cm^−2^) for 5 min and then incubated with the ROS probe DCFH‐DA and Hoechst. Scale bar = 50 µm. B) Fluorescence images of FDA/PI double‐stained cells. Cells were treated with ICG (12 µg mL^−1^) or Nb‐Ftn@ICG (equivalent to 12 µg mL^−1^ free ICG). Laser irradiation was applied to the cells indicated in the figure. Scale bar = 100 µm. C) Cell viability detected by MTT after treatment with various formulations with or without laser irradiation (n = 3). D) Intracellular ATP detected with luciferin‐based ATP assay kits. Cells were incubated with ICG (12 µg mL^−1^) or Nb‐Ftn@ICG (equivalent to 12 µg mL^−1^ free ICG) with or without laser irradiation. E) Detection of ATP released from cells after treatment with Nb‐Ftn@ICG (equivalent to 12 µg mL^−1^ free ICG) and laser irradiation for different time. F,G) Immunofluorescence assays of HMGB1 release F) and CRT exposure G). CLSM images were recorded on cells treated with ICG (12 µg mL^−1^) or Nb‐Ftn@ICG (equivalent to 12 µg mL^−1^ free ICG) and laser irradiation. HMGB1 and CRT were labeled with anti‐HMGB1 antibody and anti‐CRT antibody, respectively, and nuclei were stained with Hoechst 33342 (blue). Scale bar = 20 µm. H) Schematic description of the detection of DC maturation after co‐culture with cells pretreated with ICG (12 µg mL^−1^) or Nb‐Ftn@ICG (equivalent to 12 µg mL^−1^ free ICG) and laser irradiation. I) Percentage of mature DC cells (CD80^+^CD11c^+^) after different treatments. I: PBS, II: ICG, III: Nb‐Ftn@ICG, L: laser irradiation: 808 nm, 1 W cm+^2^, 5 min. Data are presented as the mean ± SD, n = 3.

As Nb‐Ftn@ICG and ICG can generate ROS in vitro upon laser irradiation, we measured the intracellular ROS generation induced by Nb‐Ftn@ICG and ICG using a cellular ROS probe DCFH‐DA. DCFH‐DA is a non‐fluorescent molecule that can convert to fluorescent DCF after reacting with ROS, thereby allowing monitoring ROS change in cells. The result showed that, without laser irradiation, negligible ROS was produced in cells incubated with Nb‐Ftn@ICG or ICG (**Figure** **3**A; Figure S6, Supporting Information). Upon 808 nm laser irradiation, intense ROS signals were detected in cells treated with Nb‐Ftn@ICG, while only weak ROS signals were observed in cells treated with ICG. This result is consistent with the increased cellular uptake of Nb‐Ftn@ICG relative to free ICG (Figure S3, Supporting Information).

Next, the dark‐ and photo‐cytotoxicity of various agents was analyzed via a live‐dead cell assay using CLSM on B16F10 cells using live cell probe FDA (green dye) and dead cell probe PI (red dye). It was clearly seen that ICG and Nb‐Ftn@ICG did not exhibit dark‐cytotoxicity. Upon laser irradiation, cells treated with Nb‐Ftn@ICG nearly completely died (only some cells at the edge of the laser spot suffered less damage), while only a partial number of cells treated with ICG died (Figure 3B; Figure S7, Supporting Information), indicating the significantly higher photo‐cytotoxicity of Nb‐Ftn@ICG than free ICG. This result was also confirmed by the quantified cell viability measurement. MTT assay showed that, upon laser irradiation, Nb‐Ftn@ICG inhibited the proliferation of tumor cells much more efficiently than ICG (Figure 3C).

### 2.6 Nb‐Ftn@ICG Modulates the Immunogenicity of Tumor Cells In Vitro

Photothermal therapy (PTT) is able to induce immunogenic cell death (ICD) of tumor cells, resulting in the release of DAMPs from dying cells, such as ATP and high mobility group protein 1 (HMGB1) release and calreticulin (CRT) exposure on the cell surface.^[^ ^6^, ^18^ ^]^ The released ATP acts as a “find me” signal, which subsequently causes cytokine production by DCs.^[^ ^19^ ^]^ Therefore, we measured the ATP level in the cells after laser irradiation. The result showed that the intracellular ATP level dropped considerably in the cells treated with Nb‐Ftn@ICG and laser irradiation, and less ATP release was observed in the cells treated with free ICG (Figure 3D). Meanwhile, the extracellular ATP level increased significantly at 8 h post‐PTT, followed by a gradual decrease after 8 h (Figure 3E).

ICD‐induced HMGB1 release from dying cells can trigger inflammation by attracting various immune cells and causing DC maturation.^[^ ^19^, ^20^ ^]^ By using a red dye‐labeled antibody, the localization of HMGB1 can be detected via an immunofluorescence assay with CLSM imaging. Without laser irradiation, high fluorescence intensity was observed mostly in the nuclei; after laser irradiation, the fluorescence significantly decreased in the nuclei of the cells treated with Nb‐Ftn@ICG, while less effect was observed on the cells treated with free ICG (Figure 3F; Figure S8, Supporting Information), showing prominent HMGB1 release induced by Nb‐Ftn@ICG‐based PTT.

CRT exposure is the other feature of ICD, which mediates tumor immunogenicity and delivers an “eat me” signal.^[^ ^19^, ^20^, ^21^ ^]^ CLSM imaging showed that CRT was significantly exposed on cells after incubation with Nb‐Ftn@ICG and irradiation with an 808 nm laser, while a weaker fluorescent signal was observed in cells treated with ICG+L (Figure 3G; Figure S9, Supporting Information). Cells without laser excitation did not show detectable fluorescent signal corresponding to CRT. Collectively, Nb‐Ftn@ICG induced more effective ICD through PTT, leading to HMGB1 and ATP release and CRT exposure. This result is consistent with the greater effectiveness of the Nb‐Ftn nanoplatform in terms of the enhanced cellular uptake, photothermal effect and ROS generation.

DC maturation, a feature of immunotherapy facilitated by DAMP release,^[^ ^22^ ^]^ was analyzed by incubating immature bone marrow‐derived dendritic cells (BMDCs) and tumor cells pretreated with different formulations (Figure 3H). The percentage of mature BMDCs (CD80^+^/CD11c^+^) was measured after 24 h of treatment using flow cytometry. The treatment with ICG or Nb‐Ftn@ICG in the dark moderately increased the percentage of mature DCs from 14% to 17% and 30%, respectively; upon laser irradiation, the percentage of mature DCs increased to 44% and 48%, respectively (Figure 3I; Figure S10, Supporting Information). Measuring secretion of proinflammatory cytokines, the representative hallmark of immune responses upon DC maturation, indicated that the treatment of Nb‐Ftn@ICG + L significantly increased the extracellular IL‐6 level relative to the treatment of PBS or Nb‐Ftn@ICG (Figure S11, Supporting Information), demonstrating the cytokines release from matured DCs induced by the treatment of Nb‐Ftn@ICG + L. Together with the effects of ATP release, HMGB1 release and CRT exposure, these results provided robust evidence that the nanoconjugate can induce ICD effects for promoting cancer immunotherapy.

### 2.7 NIR‐II Photo Imaging and PTT Effect In Vivo

**Figure 4:**
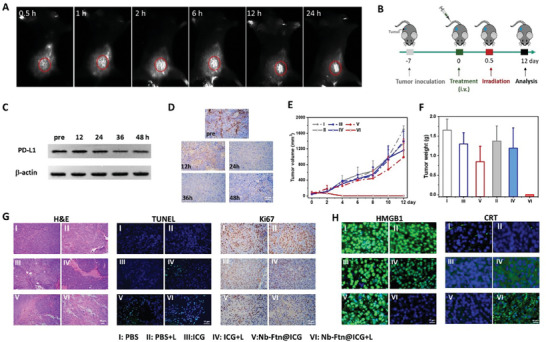
In vivo therapeutic efficacy of Nb‐Ftn@ICG against primary tumors in B16F10 tumor‐bearing mice. A) In vivo NIR‐II imaging of tumor‐bearing mice after intravenous injection of Nb‐Ftn@ICG (equivalent to 2.25 mg k^−1^g free ICG). The red circles indicate the location of the tumors. B) Schematic illustration of the time schedule of tumor treatment. Mice were i.v. injection of PBS, ICG or Nb‐Ftn@ICG (day 0 treatment) following laser irradiation after 0.5 days. C,D) Western blot assay C) and immunohistochemistry analysis D) of PD‐L1 levels in the tumors excised from mice at different times after a single injection of Nb‐Ftn (10.0 mg k^−1^g, equivalent to 0.27 nmol per 20 g mouse) (*n *= 3) without laser irradiation. Scale bar = 50 µm. E) Tumor growth curve over 12 days (n = 5). F) Weight of tumors on day 12 (*n* = 5). G) Representative H&E staining images, TUNEL images, and Ki67‐stained images of tumor tissues after various treatments. Scale bar = 50 µm. H) Representative immunofluorescence images of HMGB1 release and CRT translocation of cells in the tumors excised from mice 12 h after various treatments. Scale bar = 20 µm. Laser irradiation: 808 nm, 1 W cm^−2^, 5 min.

As ICG can act as a NIR‐II dye, the biodistribution of Nb‐Ftn@ICG can be traced using NIR‐II fluorescence imaging. The fluorescence of Nb‐Ftn@ICG can be clearly detected in the mice shortly after administration. While gradual clearance of the fluorescence was observed throughout the body, the accumulation of Nb‐Ftn@ICG in tumors was observed by intensified fluorescence (**Figure** **4**A). By comparison, free ICG can be cleared rather rapidly after administration (Figure S12, Supporting Information). This result confirmed the tumor targeting ability of the Nb‐Ftn nanoplatform, which led to significant tumor accumulation and retention.

The tumor accumulation of Nb‐Ftn@ICG is expected to enhance the PTT effect in vivo. Monitoring temperature of tumors indicated that, after 5 min of laser irradiation (1 W cm^−2^), the temperature at tumor site elevated by 19.6 °C in the mice treated with Nb‐Ftn@ICG, compared to 10.5 °C in those treated with free ICG and 6.7 °C treated with PBS (Figure S13, Supporting Information). Thus, Nb‐Ftn@ICG possesses highly potent PTT property in vivo by tumor targeting.

### 2.8 The Downregulation of PD‐L1 In Vivo

Encouraged by the down‐regulation of PD‐L1 induced by Nb‐Ftn at the cellular level, the effect of the nanoplatform on the PD‐L1 level in vivo was evaluated. The tumor‐bearing mice were treated with *i.v*. injection of Nb‐Ftn (10.0 mg kg^−1^g, 0.27 nmol per 20 g mouse), and the tumors were excised at different times post‐administration. Western blot and immunohistochemistry analyses indicated that the PD‐L1 level decreased in a time‐dependent manner (Figure 4C,D; Figure S14, Supporting Information). This result confirmed that the Nb‐Ftn nanoplatform can degrade PD‐L1 in tumor cells in vivo.

### 2.9 In vivo Therapeutic Efficacy Against Primary Tumors

The therapeutic efficacy of Nb‐Ftn@ICG‐mediated PTT was assessed in B16F10 tumor‐bearing mice. The mice were randomly allocated to six groups, namely, PBS, PBS + L, ICG, ICG + L, Nb‐Ftn@ICG and Nb‐Ftn@ICG + L, where L denotes laser irradiation. The treatment regimen is shown in Figure 4B. The tumor growth curve during treatment is depicted in Figure 4E, and the weight and images of tumors excised from mice on day 12 are illustrated in Figure 4E,F and Figure S15 (Supporting Information). The result demonstrated that tumors were ablated in the Nb‐Ftn@ICG‐treated mice with PTT, while ICG treatment only slightly suppressed tumor growth after receiving PTT. In addition, H&E staining and TUNEL assay revealed that most apoptotic bodies were detected in the tumors of the mice in the “Nb‐Ftn@ICG + L” group (Figure 4G). Meanwhile, the Ki67 assay indicated that the tumors in the “Nb‐Ftn@ICG + L” group manifested the lowest number of Ki67‐positive cells (brown‐yellow). These results revealed the highly potent antitumor activity of Nb‐Ftn@ICG with PTT treatment.

The systemic toxicity of Nb‐Ftn@ICG was subsequently assessed. Monitoring the body weight showed that no significant changes occurred during treatments, suggesting low toxicity of Nb‐Ftn@ICG with PTT treatment (Figure S16, Supporting Information). The measurement of major blood biochemical parameters (alkaline phosphatase, aspartate aminotransferase, alanine aminotransferase, blood urea nitrogen and creatinine) indicated that no obvious differences were detected in the mice treated with Nb‐Ftn@ICG (Figure S17, Supporting Information). Additionally, pathological analysis revealed no significant toxicity toward major organs extracted from tumor‐bearing mice treated with Nb‐Ftn@ICG (Figure S18, Supporting Information). Taken together, these results demonstrated the favorable biocompatibility of Nb‐Ftn@ICG.

### 2.10 DAMP Release and DC Maturation In Vivo

Next, the PTT effect of Nb‐Ftn@ICG on the induction of immunogenic phenotypes in vivo was investigated. B16F10 tumor‐bearing mice were treated with different formulations and then subjected to laser irradiation for 5 min. Tumor tissues were excised 12 h post‐PTT, and the levels of HMGB1 and CRT in the tumors were analyzed via immunofluorescence assay. A considerable decrease of HMGB1 levels (green fluorescence) was observed in the tumors of the mice treated with “Nb‐Ftn@ICG + L” (Figure 4H, left portion), which is consistent with the in vitro results (Figure 2F). In line with this result, immunofluorescence staining results revealed an elevation in CRT exposure in the tumors of the mice treated with Nb‐Ftn@ICG+L (Figure 4H, right portion). Subsequently, the percentage of mature DCs in tumors was measured on day 3 following treatment with different formulations. Flow cytometry analysis demonstrated that the ratio of mature DCs (CD86^+^/CD80^+^) increased considerably in the tumors of the mice that received “Nb‐Ftn@ICG + L” treatment (to 8.9%), while “ICG + L” treatment led to a minor increase of DC maturation level (to 1.45%) (Figure S19, Supporting Information). Without laser irradiation, treatment with Nb‐Ftn@ICG or free ICG had a negligible effect on DC maturation (1.0–1.2%), and the ratios were similar to those of the control group treated with PBS.

### 2.11 Distal Effect and Anti‐Metastasis Activity

**Figure 5:**
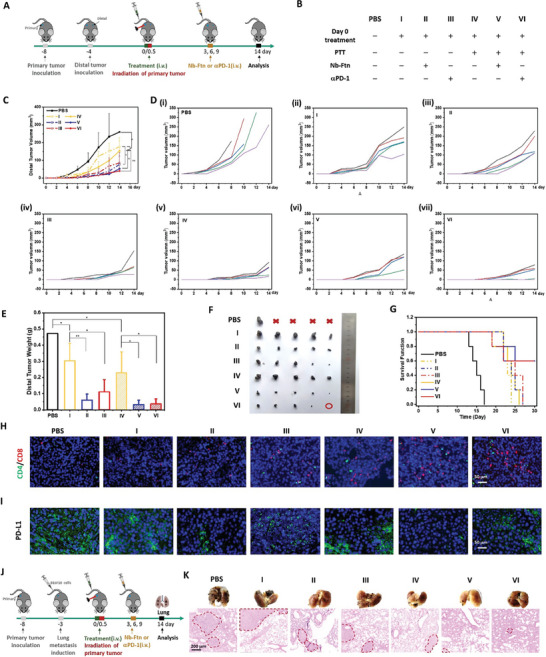
In vivo assays of distal tumor suppression and anti‐metastasis. A,B) Schema of the anti‐distal tumor treatment. Mice were randomly divided into seven groups and inoculated with primary tumors and distal tumors on the day −8 and the day −4, respectively. Mice received i.v. injection of PBS or Nb‐Ftn@ICG (groups I–VI) on the day 0. The treatments of PTT (day 0.5) and following immunotherapy (days 3, 6 and 9) of each group are listed in Figure 5B. C,D) The growth curve (C) and their spaghetti curves (D) of distal tumors after various treatments over 14 days (n = 5). E) Weight of distal tumors on day 14 (*n* = 5). F) Photographs of excised distal tumors from tumor‐bearing mice after different treatments (*n* = 5). Red X indicates that the mice died before day 14. The red circles indicate that the tumor was completely ablated. G) Kaplan–Meier survival curve of the tumor‐bearing mice. H) Immunofluorescence analysis of the intra‐tumoral infiltration of CD4 and CD8 T cells in the tumor‐bearing mice after different treatments. CD4^+^ (green) and CD8^+^ (red) T cells were stained with anti‐CD4 antibody and anti‐CD8 antibody, respectively. Scale bar = 50 µm. I) Immunofluorescence analysis of distal tumors excised from mice after different treatments (*n* = 5). PD‐L1 was stained with an anti‐PD‐L1 antibody (labeled with green dye) and nuclei were stained with DAPI (blue). Scale bar = 50 µm. J) Schema of the anti‐metastasis study. Mice were randomly divided into seven groups and inoculated with primary tumors on the day −8 and i.v. injected with tumor cells on the day −3. The following treatments from day 0 were the same as the schedule listed in Figure 5B. K) Tumor images (top portion) and representative H&E staining images (bottom portion) of metastatic nodules in the lung. Scale bar = 200 µm. Laser irradiation: 808 nm, 1 W cm^−2^, 5 min.

Encouraged by the promising immune response of the Nb‐Ftn@ICG nanoplatform, including tumor targeting, blockade of the PD‐1/PD‐L1 interaction, PD‐L1 degradation, and DAMP release in vitro and in vivo, the abscopal antitumor effect was further evaluated in a bilateral B16F10 tumor‐bearing mouse model. The distal tumors (on the right side) were inoculated 4 days after planting the primary tumors (on the left side), and drug treatment and PTT were administered after another 4 days (**Figure** **5**A). Laser irradiation was applied to primary tumors, while distal tumors were obscured from irradiation. The mice also received additional treatment with Nb‐Ftn in order to enhance the therapeutic effect on distal tumors (Figure 5A,B). In this circumstance, the comparison of anti‐PD‐1 antibody (αPD‐1) was used to evaluate the immunotherapeutic efficacy of the Nb‐Ftn platform. As expected, the primary tumors were ablated by the initial PTT, after which the growth of distal tumors was monitored by measuring the tumor volumes (Figure 5C,D). After 14 days of treatment, the mice were sacrificed, the tumor weights were measured, and tumor images were recorded (Figure 5E,F).

The result demonstrated that, even without PTT, single treatment with Nb‐Ftn exhibited a moderate inhibitory effect on distal tumor growth by reducing the tumor weight by 35 ± 21% (group I) (Figure 5E,F). More significant inhibition of distal tumors was observed in the mice that received follow‐up treatment with Nb‐Ftn or αPD‐1, resulting in tumor reduction by 83 ± 8% (group II) and 76 ± 16% (group III), respectively. This result suggests that Nb‐Ftn can act as an effective ICB agent, considering that a lower dosage of Nb‐Ftn (0.27 nmol) than αPD‐1 (1.0 nmol) was used in the assay. Compared to the single injection of Nb‐Ftn (group I), PTT (group IV) did not exhibit better suppression of the growth of distal tumors, suggesting that PTT alone did not lead to a sufficient immune response. Notably, the combination of PTT with immunotherapy of αPD‐1 or Nb‐Ftn significantly inhibited the growth of distal tumors (Figure 5C,F), showing the importance of combinational treatments in immunotherapy.

The mechanism of the enhanced immunotherapy of the Nb‐Ftn nanoplatform was further investigated by assessing T‐cell infiltration in distal tumors. As a result of DC maturation and blockade of the PD‐1/PD‐L1 interaction, the number of CD4^+^ and CD8^+^ T‐cells clearly increased after PTT with Nb‐Ftn@ICG, followed by additional treatment Nb‐Ftn or αPD‐1 (Figure S20, Supporting Information), confirming the activation of T cell‐mediated immune response. Meanwhile, immunofluorescence measurement showed that the populations of CD4/CD8‐positive T‐cells in distal tumors were enhanced by the combinational treatment of PTT with αPD‐1 or Nb‐Ftn (Figure 5H), which promoted cell autoimmunity. Immunofluorescence assay also revealed that the Nb‐Ftn nanoplatform effectively downregulated the PD‐L1 level in distal tumors (Figure 5I), in consistence with the degradation of PD‐L1 observed in the cellular assays. By comparison, treatment with αPD‐1 did not cause detectable alteration in PD‐L1 levels in the tumor. Together with the degradation of PD‐L1 by the Nb‐Ftn platform, the combination treatment of Nb‐Ftn or αPD‐1 would further promote immunotherapy by reducing the PD‐L1/PD‐1 interaction‐mediated immunosuppression. Consequently, this combination therapy using PTT with Nb‐Ftn or αPD‐1 effectively inhibited tumor growth and prolonged the survival of mice (Figure 5G). There were no significant changes in body weight or observable damage to major organs in mice treated with the combination therapy (Figures S23 and S24, Supporting Information).

In addition, the T‐cell alterations caused by the treatment in tumors were assessed using flow cytometry on the bilateral tumor‐bearing mouse model by measuring CD3^+^, CD4^+^, CD8^+^ and CD69^+^ T‐cell levels in the distal tumors. Results demonstrated that the treatment of Nb‐Ftn@ICG + L plus Nb‐Ftn (corresponding to the group V in Figure 5B) led to significant increase in the ratios of CD4^+^/CD3^+^ and CD8^+^/CD3^+^ T cells in the distal tumors 5 days post‐administration (Figure S21, Supporting Information), indicating the enhanced T‐cell infiltration. In comparison, the treatment of Nb‐Ftn@ICG without laser irradiation (corresponding to the group I in Figure 5B) did not cause detectable changes of CD4^+^/CD3^+^ and CD8^+^/CD3^+^ ratios of T cells at this stage, confirming the PTT‐promoted T‐cell infiltration. On the other hand, moderate activation of T cells was observed on treatment of Nb‐Ftn@ICG plus Nb‐Ftn after longer time (8 days post‐administration), while more significant effect was observed with PTT treatment (Nb‐Ftn@ICG + L plus Nb‐Ftn) (Figure S22, Supporting Information). In consistence with ICD effect of these formula, the observation on the T‐cell alteration in distal tumors confirmed that the degradation of PD‐L1 and blockade of the PD‐1/PD‐L1 interaction synergistically contributed to the infiltration and activation of T cells.

Considering that the combination of PTT and Nb‐Ftn treatment would induce systemic immune activation, a more aggressive lung metastatic model was established by i.v. injection of tumor cells 5 days after implantation of the primary tumor, by then the tumor appeared in proper size on the right back of the mice. The lung images and H&E staining clearly showed that, 17 days after i.v. injection of tumor cells, a large number of metastatic nodules were observed in the lungs of mice in the PBS control group (Figure 5K). The mice in the treatment groups that received Nb‐Ftn@ICG and laser irradiation exhibited noticeably fewer nodules compared to the corresponding non‐light‐exposure group. Importantly, combination therapy (PTT and Nb‐Ftn or αPD‐1) displayed minimal observable nodules, which effectively inhibited tumor infiltration, demonstrating a very favorable therapeutic outcome of the PTT and ICB method.

Multivalent antibodies provide high chances for their paratopes binding to targets with decreased dissociation rates, which could increase the retention to receptors and enhance the biological response to the target proteins. To achieve this goal, a variety of approaches have been designed to multimerize antibodies. Ferritin nanocage, a natural iron storage protein assembled by 24 subunits, offers a unique scaffold for construction of functional protein delivery systems due to its prominent stability, biocompatibility and pharmacokinetic properties. Antibody‐ferritin conjugates can achieve highly specific targeting for delivering therapeutic and diagnostic agents.^[^ ^23^ ^]^ Recently, ferritin was used as a frame to construct a nanoparticle vaccine, which showed potent, long‐lasting, and broad‐spectrum neutralization properties.^[^ ^24^ ^]^ However, the large steric hindrance could limit the conjugation of multiple large size of conventional antibody (≈150 kDa) to ferritin (12 nm sphere) and adversely affect the conformation, stability, and binding capacity of the antibody.

Nanobody (≈15 kDa) is the smallest natural antibody that possesses high antigen binding affinity similar to conventional antibody. The small size of nanobody endows it unique properties for convenient construction of multivalent formations with minimal complexity. On the other hand, the disadvantage of rapid clearance of nanobody can be overcome by multivalent construction.^[^ ^11a^ ^]^ Hence, the conjugation of nanobody‐ferritin takes advantages of two components to enhance the therapeutic effect in immunotherapy. In addition to the assembly structure for multimerization of nanobody, ferritin is also an ideal drug carrier due to its cavity structure.

In this work, we conjugated an anti‐PD‐L1 nanobody to the outer surface of ferritin through specific ligation, forming a tumor‐targeting Nb‐Ftn nanoplatform. Nb‐Ftn possesses a hydrodynamic diameter of ≈28 nm, which prevents rapid renal clearance (typical for spherical nanoparticles < 5 nm in diameter) and filtration by liver and spleen systems (for spherical nanoparticles > 150 nm in diameter).^[^ ^25^ ^]^ In addition, the encapsulation of the photosensitizer ICG in the ferritin cavity enables the PTT treatment of Nb‐Ftn@ICG and allows NIR‐II imaging of the drug distribution and tumor accumulation. The nanobody was conjugated to ferritin after the encapsulation of ICG, which avoids the dysfunction of the nanobody during the drug loading process.

As ferritin is assembled by 24 subunits, multiple nanobody molecules can be conjugated to the protein cage, forming a structure that is similar to multivalent nanobodies. It is well‐known that multivalent antibodies provide high chances for their paratopes binding to targets with decreased dissociation rates, which could increase the retention to receptors and enhance the biological response to the target proteins.^[^ ^26^ ^]^ In addition to the increased stability, target binding and prolonged in vivo circulation, one important feature is that Nb‐Ftn can induce degradation of PD‐L1, which could result in more efficient PD‐1/PD‐L1 blockade. PD‐L1 degradation is a potential strategy to enhance the effectiveness of immunotherapy.^[^ ^27^ ^]^ Therefore, several approaches, including antibody‐based proteolysis targeting chimeras (PROTACs)^[^ ^28^ ^]^ and lysosome‐targeting chimeras (LYTACs),^[^ ^17a^ ^]^ have been recently designed to enhance PD‐1/PD‐L1‐based immunotherapy by PD‐L1 targeting and degradation. It has been found that the multimerization of nanobody or antibody leads to more efficient downregulation of receptor proteins than monovalent ones.^[^ ^29^ ^]^ In this work, the Nb‐Ftn nanoplatform offers a new approach to degrade PD‐L1 both in vitro and in vivo, resulting in efficient PD‐1/PD‐L1 blockade. By comparison, monovalent PD‐L1 nanobody did not exhibit the function of degradation of the target protein. Moreover, this multivalent Nb‐Ftn can achieve a similar immunotherapeutic response at a lower molar concentration than conventional antibody. These results highlight the significance of multivalent nanobody through ferritin conjugation.

Systemic immune response is extremely important for immunotherapy, as metastasis is the key cause of tumor‐related death. Immunotherapy brings a new opportunity for complete ablation of metastatic tumors. Combination with additional therapies, such as radio‐therapy or photothermal therapy, could synergistically enhance tumor immunogenicity by relieving immunosuppression and activating T‐cell functions. On the one hand, the Nb‐Ftn nanoplatform designed in this work can enhance tumor immunogenicity by PTT‐mediated ICD of tumors; on the other hand, Nb‐Ftn can inhibit the interaction of PD‐1/PD‐L1 and downregulate PD‐L1 levels. The synergistic PTT and immune checkpoint inhibition of Nb‐Ftn resulted in enhanced tumor killing and effective immune activation. Considering the low toxicity and biodegradability of protein‐based nanoparticles, the FDA‐approved photosensitizer, and the biocompatible assembly procedure, this Nb‐Ftn@ICG nanoplatform exhibited high potential for further clinical development. It can be anticipated that this nanobody‐ferritin conjugation strategy can be applied for more functional designs by changing the targeting nanobody and payload in the ferritin cavity.

## 3 Conclusion

In summary, we constructed a nanobody‐ferritin nanoplatform (Nb‐Ftn) for photothermal therapy‐enhanced immunotherapy. Nb‐Ftn was generated by site‐specific conjugation of an anti‐PD‐L1 nanobody (Nb) to the exterior surface of ferritin (Ftn). The display of Nb molecules on the Ftn nanocage results in a multivalent nanobody conjugate that can effectively target PD‐L1‐positive tumor cells and disrupt the function of PD‐L1. This multivalent Nb‐Ftn nanoplatform can act as a potent immune checkpoint blockade (ICB) agent by blocking the PD‐1/PD‐L1 interaction; in addition, it also downregulates the PD‐L1 level of tumor cells by inducing lysosomal degradation. Therefore, Nb‐Ftn can induce immune responses more efficiently than the conventional anti‐PD‐L1 antibody (αPD‐L1). Moreover, the ferritin nanocage allows encapsulation of the FDA‐approved photosensitizer ICG, generating the tumor‐targeting PTT agent Nb‐Ftn@ICG. Nb‐Ftn demonstrated enhanced PTT and PDT efficacy both in vitro and in vivo relative to free ICG, which is associated with the tumor‐targeting and endocytosis‐induced tumor accumulation of Nb‐Ftn. The PTT effect of Nb‐Ftn@ICG induces immunogenic cell death (ICD), manifested by the release of ATP and HMGB1, as well as the exposure of CRT, further enhancing the effect of immunotherapy. Together with the blockade effect and degradation of PD‐L1, the combination therapy leads to effective ablation of the primary tumor, suppression of distal tumors and inhibition of metastasis.

## Conflict of Interest

The authors declare no conflict of interest.

## Supporting information

Supporting Information

## Data Availability

The data that support the findings of this study are available in the supplementary material of this article.
