# Supplementary material for: Enhancing Tumor Immunotherapy by Multivalent Anti‐PD‐L1 Nanobody Assembled via Ferritin Nanocage
Source: Adv Sci (Weinh). 2024 Mar 16;11(20):2308248. doi: 10.1002/advs.202308248 (PMC11132087; doi:10.1002/advs.202308248)

ADVANCED SCIENCE
Open Access

Supporting Information

for *Adv. Sci.*, DOI 10.1002/advs.202308248

Enhancing Tumor Immunotherapy by Multivalent Anti-PD-L1 Nanobody Assembled via Ferritin Nanocage

Manman Liu, Duo Jin, Wenxin Yu, Jiaji Yu, Kaiming Cao, Junjie Cheng, Xiaohu Zheng, Andrew Wang and Yangzhong Liu**

Supporting information

for

Enhancing Tumor Immunotherapy by Multivalent anti-PD-L1 Nanobody Assembled via Ferritin Nanocage

Manman Liu, Duo Jin, Wenxin Yu, Jiaji Yu, Kaiming Cao, Junjie Cheng, Xiaohu Zheng, Andrew Wang, Yangzhong Liu

Content

1. **Supplemental Experimental Procedures**

2. **Supplemental Figures**

Figure S1. SDS-PAGE analyses of the conjugation of Nb-Q with PEGylated Ftn@ICG

Figure S2. Photothermal effect of Nb-Ftn@ICG in solution 808 nm laser irradiation

Figure S3. Cellular uptake of Nb-Ftn@ICG measured with fluorescence imaging

Figure S4. Flow cytometry analysis of blockade of the PD-1/PD-L1 interaction

Figure S5. Quantification of the cellular PD-L1 level downregulated by Nb-Ftn

Figure S6. Fluorescence imaging of ROS generation in cells

Figure S7. Fluorescent imaging of live/dead cell assay

Figure S8. HMGB1 release measured with CLSM imaging

Figure S9. CRT exposure measured with CLSM imaging

Figure S10. Flow cytometry analysis of DC maturation

Figure S11. Relative cytokine levels of IL-6 in DC suspensions were detected by ELISA kits

Figure S12. *In vivo* NIR-II imaging of B16F10 tumor-bearing mice

Figure S13. *In vivo* photothermal effect of Nb-Ftn@ICG

Figure S14. Quantification of the total PD-L1 levels in tumors

Figure S15. Photographs of excised tumors from mice

Figure S16. Weights of B16F10 tumor-bearing mice during the treatments

Figure S17. Blood biochemical tests of healthy mice after Nb-Ftn@ICG treatment

Figure S18. H&E staining images of major organs

Figure S19. Flow cytometry analysis of mature DCs ($CD86^+$/$CD80^+$) in tumors

Figure S20. Immunohistochemistry analysis of distal tumors

Figure S21. Representative flow cytometry plots of T cells in tumors 5 days post various treatments

Figure S22. Representative flow cytometry plots of active T cells in tumors 8 days post various treatments

Figure S23. Weights of B16F10 tumor-bearing mice during the treatment

Figure S24. Representative H&E staining images of major organs of bilateral B16F10-bearing mice

3. **Full Images of Western Blotting**

1. Supplemental Experimental Procedures

Materials

MAL-PEG5k-NH_2 (PS2-NM-5K) was purchased from Ponsure Biotechnology (Shanghai, China). Indocyanine green (ICG), 3-[4,5-dimethylthiazol-2-yl]-2,5-diphenyltetrazolium (Hoechst 33342), fluorescein isothiocyanate (FITC) and 1,3-diphenylisobenzofuran (DPBF) were obtained from Aladdin Reagent (Shanghai, China). The anti-PD-1 antibody (mouse) used in vivo was purchased from BioXCell. CD86-FITC, CD80-PE, and CD11c-APC were purchased from Biolegend (San Diego, CA, USA). Antibodies against PD-L1 and β-actin for western blot assay, anti-HMGB1 antibody, anti-CRT antibody and Alexa Fluor® 555-labeled goat anti-rabbit IgG for immunofluorescence were obtained from Abcam (Shanghai, China). Lyso-Tracker Green, and Reactive Oxygen Species assay kits were purchased from Beyotime Biotechnology (Shanghai, China). Microbial transglutaminase (mTGase) was provided by Yiming Biological (China).

Protein expression and purification

mPD-1 (extracellular domain of the murine PD-1) was expressed in *E. coli* cells. The cDNA sequence of mPD-1 came from the literature,[30] and the plasmid was constructed with a fusion of GB1-tag including 6×His residues at the N-terminus of mPD-1 (GB1-mPD-1). The plasmid was synthesized by Tsingke Biotechnology Co., Ltd. (Beijing, China) using whole genetic synthesis. Then, the plasmid was transformed into *E. coli* cells (Gold), and cells grew on a Luria-Bertani (LB) plate with ampicillin. Cells from a single clone were incubated overnight in 4.5 mL LB medium within 1 mg/mL Ampicillin. Cells were transformed into 1 L LB medium until the OD_{600} reached to approximately 0.8. Proteins expression was induced by IPTG (0.4 mM) for 4 h at 37°C. Then, the cells were harvested, and the cell pellets were resuspended in 20 mL of buffer (50 mM Tris, 150 mM NaCl, pH 8.0). Next, the cells were sonicated on ice and subsequently centrifuged to remove *E. coli* debris. The supernatant was loaded onto a Ni-NTA column and washed with washing buffer (50 mM Tris, 150 mM NaCl, 20 mM imidazole (IMD), pH 8.0). The protein was eluted with elution buffer (50 mM Tris, 150 mM NaCl, 500 mM IMD, pH 8.0), and the flow through was collected for further purification by size-exclusion chromatography (SEC) using a Superdex 75 Column (GE healthcare Life Sciences, Pittsburgh, PA, USA). The concentration of the purified protein was measured by BCA assay.

The anti-PD-L1 (mouse) nanobody with a Q-tag (Nb-Q) was overexpressed in *E. coli* cells according to the literature method.[13, 31] The plasmid containing the gene sequence of an anti-PD-L1 Nb and a Q-tag was constructed by genetic engineering. The cDNA sequence of anti-PD-L1 Nb was obtained from the literature.[32] The expression and purification of heavy chain ferritin (Ftn) were conducted according to our previous work.[13]

The FITC labeling of proteins was achieved by the addition of a 10-fold molar ratio of FITC (dissolved in DMSO) to mPD-1 in PBS (pH 8.5) and incubation for 12 h under magnetic stirring at 4°C. The excess FITC was removed by dialysis (3 kDa cutoff). Then, FITC-labeled mPD-1 was further purified using desalting columns (GE Healthcare) in PBS (pH 7.2–7.4).

Cell lines

B16F10 cells were obtained from Guangzhou Cellcook Biotechnology. Cells were maintained in RPMI-1640 medium supplemented with 10% FBS and penicillin/streptomycin in a humidified atmosphere containing 5% CO_2 at 37°C.

Animals

Six-eight-week-old female C57BL/6 mice were purchased from Vital River Laboratory Animal Technology Co., Ltd. (Beijing, China). All animal experiments were conducted strictly under the guidance of the Committee of the Animal Experiment Center of the University of Science and Technology of China (Hefei, China) and the Regulations on the Administration of Laboratory Animal Affairs with the assigned accreditation number PXHG-LYZ2021041519.

Preparation of Nb-Ftn@ICG

PEGylation of Ftn: PEGylation of Ftn was conducted by covalent conjugation of MAL-PEG_{5k}-NH_2 to the thiol group of cysteine on the surface of Ftn. Ftn (10 μM) was incubated with TCEP (720 μM) for 30 min at 37°C, and the mixture was loaded on desalting columns (GE Healthcare) to remove TCEP. The PEGylation of Ftn was achieved by incubation of purified Ftn (5 μM)

with MAL-PEG$_{5k}$-NH$_2$ (5 μM) overnight at 25°C. The unreacted PEG was removed using an ultra-centrifugal filter (100 kDa cut-off, Millipore) thrice with buffer (50 mM Tris, 150 mM NaCl, pH 8.0). The product was collected and analyzed with SDS-PAGE.

Drug Loading: PEGylated Ftn (3 mg/mL, 2 mL) was placed in a glass bottle and adjusted to pH 2.5 using 30% acetic acid. After 30 min of stirring, 0.2 mL of ICG in DMSO was added dropwise. After magnetic stirring for 30 min, the pH of the solution was tuned to 8.0 with 1 M NaOH under pH monitoring. The PEGylated Ftn reassembled during 2 h of stirring, and the resulting solution was centrifuged for 10 min to remove precipitates. Most of the free ICG and DMSO were removed from the supernatant by dialysis (50 kDa cutoff). The obtained PEGylated Ftn@ICG was purified again with a desalting column. The concentration of protein was measured with a BSA assay, and the content of ICG loaded in the protein was quantified by the absorbance of ICG using a UV-vis spectrophotometer (TU-1901, Bjpersee).

Nanobody Conjugation: The conjugation of nanobodies to PEGylated ferritin was performed with the procedures reported in our previous work.[13] In brief, PEGylated Ftn or PEGylated Ftn@ICG were mixed with Nb-Q in a series of ratios from 5:1 to 30:1, which gave different conjugation ratios of Nb to Ftn. This conjugation reaction was catalyzed by mTGase (1 mg/mL) for 1 h in PBS buffer at 25°C. The product was analyzed with SDS-PAGE and further purified through size exclusion chromatography (SEC).

Experiments with ICG were performed in the dark.

Characterizations

SEC analysis: SEC was performed on an ÄKTA purifier system equipped with a Superose 6 Increase 10/300 GL (Tricorn) column (GE Healthcare, 29-0915-96) to analyze the retention volumes of Nb-Ftn@ICG, PEGylated Ftn@ICG and Ftn. The flow rate was set at 0.5 ml/min, and UV detection at 280 nm was used.

Dynamic light scattering (DLS) analysis: Hydrodynamic diameters of Nb-Ftn@ICG, Nb-Ftn, PEGylated Ftn@ICG and Ftn were detected by DLS (Zetasizer μV, Malvern). For the stability analysis, the hydrodynamic diameters of Nb-Ftn and Nb-Ftn@ICG were detected at different time points over 120 h.

Transmission electron microscope (TEM) photography: 1 mg/mL Nb-Ftn@ICG and Ftn were placed onto carbon-coated copper grids and negatively stained using phosphotungstic acid. After drying, the copper grids were imaged on a Tecnai G2 Spirit BioTWIN electron microscope at 120 kV.

Evaluation of photothermal efficacy and ROS generation

Photothermal effect in solution: A series of concentrations of Nb-Ftn@ICG (equivalents to 0 - 30 μg/mL ICG) in solution was irradiated with an 808 nm laser at different powers for 5 min. The temperature changes of Nb-Ftn@ICG were recorded by a thermocouple microprobe (DT-847UD, ATEST Thermometer). The temperature change of free ICG in aqueous solution was also detected for comparison.

ROS generation: 1,3-diphenylisobenzofuran (DPBF) was used as a probe for detecting ROS. Briefly, 1 mL Nb-Ftn@ICG or ICG (5 μg/mL of ICG) was mixed with DPBF, and solutions were irradiated with an 808 nm laser (1.0 W/cm^2). Then, the absorbance of DPBF was recorded using a UV–vis spectrophotometer (TU-1901, bjpersee) within 5 min. A DPBF solution in the absence of ICG was used as a reference.

Cell uptake and subcellular distribution. 1×10^5 B16F10 cells were seeded in 12-well plates. After growing overnight, the cells were incubated with Nb-Ftn@ICG (equivalent to 60 μM free ICG) or ICG (60 μM) for 0.5, 1 or 2 h. Then, the cell culture medium was replaced with fresh medium containing Hoechst 33342 for nuclear staining. After another 10 min incubation, the cells were washed with fresh PBS and then imaged by fluorescence imaging (IX73, Olympus).

For the lysosomal co-localization assay, 5×10^4 B16F10 cells were seeded in confocal dishes. After grow overnight, the cells were cultured with RPMI 1640 medium containing Nb-Ftn@ICG (0.6 μM) for 1 or 2 h, then, the cells were incubated in RPMI 1640 medium with Lyso-Tracker Green and Hoechst 33342 for 20 - 30 min. Then, cell fluorescence images were recorded on a confocal laser scanning microscope (LSM880, Zeiss).

Intracellular ROS generation. Cells were seeded in 6-well plates at a density of 1.5 × 10^5 cells/well. After growing for 24 h, cells were cultured with medium containing free ICG or Nb-Ftn@ICG (equivalent to 10 μg/mL ICG) for 2 h. Then the culture medium was replaced with fresh medium, and the cells were irradiated with an 808 nm laser (1.0 W/cm^2) for 5 min. Then, the cells were incubated with fresh medium containing DCFH-DA for 20 min, followed by incubation in fresh medium containing Hoechst 33342 for 15 min. After PBS washing, the cells were subjected to fluorescence imaging on a fluorescence microscope (IX73, Olympus).

Cytotoxic assays

MTT assay: Cells were seeded in 96-well plates at a density of 5 × 10^3 cells/well. After incubation for 24 h, the cells were incubated with fresh medium containing concentrations of ICG or Nb-Ftn@ICG for 2 h, followed by 808 nm laser irradiation (1.0 W/cm^2) for 5 min. After incubation for another 24 h, the culture medium was replaced with 100 μL of MTT solution, and the cells were incubated for another 4 h. Viable cells reduced the yellow tetrazolium salt (MTT) into blue-purple formazan crystals. Then, the crystals were dissolved in 150 μL of DMSO, and the absorbance of the products was detected at 490 nm with a Bio-Rad 680 microplate reader after 15 min of shaking.

Live/Dead cell assay: Cells were seeded in 12-well plates at a density of 8 × 10^4 cells/well. After growing for 24 h, the cells were cultured with medium containing free ICG or Nb-Ftn@ICG (equivalent to 12 μg/mL ICG) for 2 h, followed by 808 nm laser irradiation (1.0 W/cm^2) for 5 min. Then, the cells were incubated for another 8 h. After three washes with PBS, the cells were stained with FDA (10 μM) and PI (20 μM) for 20 min. The cells were washed with PBS again before fluorescence microscopic analysis (IX71, Olympus).

Detection of DAMPs release. 5 × 10^4 cells were seeded in a confocal dish. After growing for 24 h, the cells were cultured with fresh medium containing free ICG (12 μg/mL) or Nb-Ftn@ICG (equivalent to 12 μg/mL of free ICG) for 2 h, followed by 808 nm laser irradiation (1.0 W/cm^2) for 5 min. The cells were incubated for another 6 h before HMGB1 release and CRT translocation analyses.

For the HMGB1 release assay, cells were fixed with 4% paraformaldehyde, permeated with 0.1% Triton X-100, and incubated with 5% goat serum to block nonspecific binding sites. Then, the cells were incubated with anti-HMGB1 antibody, followed by incubation with Alexa Fluor® 555-labeled goat anti-rabbit IgG.

For the CRT translocation detection, cells were fixed with 4% paraformaldehyde and incubated with 5% goat serum. The cells were stained with anti-CRT antibody, and then incubated with Alexa Fluor® 555-labeled goat anti-rabbit IgG. Finally, the cells were stained with Hoechst 33342 before CLSM imaging (LSM880, Zeiss).

For ATP release detection, 5 × 10^3 cells were seeded in 96-well. After 24 h growing, the cells were treated with fresh medium containing free ICG (12 μg/mL) or Nb-Ftn@ICG (equivalent to 12 μg/mL of free ICG) for 2 h, followed by 808 nm laser irradiation (1.0 W/cm^2) for 5 min. After PTT, ATP in cell supernatants was collected at different time points (4, 8, 12, 24 h) for measuring ATP release. For detection of intercellular ATP, 5 × 10^4 cells were seeded in 12-well plates. The cells were treated in the same way as in ATP release assay. At 4 h after PTT, cells were collected and lysed. The amount of ATP in the cell lysate was detected using an ATP assay kit (S0026, Beyotime) on a luminometer (Centro LB 960, Berthold).

Cellular PD-L1 level measurement. Cells were seeded in 6-well plates at a density of 2 × 10^5 cells/well. After 24 h growing, the cells were incubated with fresh media containing various concentrations of Nb-Ftn for different time. After PBS washing, the cells were collected and lysed with RIPA lysis buffer (P0013B, Beyotime) for 30 min at 4°C. The obtained mixture was centrifuged, and the supernatant was collected. The PD-L1 levels were analyzed with western blotting.

Competitive binding assay. For CLSM measurements, 5 × 10^4 cells were seeded in a confocal dish. After 24 h growing, the cells were treated with FITC-mPD-1 (2 μM) or a mixture of FITC-mPD-1 (2 μM) and Nb-Ftn (0.2 μM) and then incubated for 20 min at 4°C. After PBS washing, the cells were stained with Hoechst 33342. Then, the cells were washed with PBS again before CLSM imaging.

For flow cytometry detection, cells were seeded in 6-well plates at a density of 1×10^5 cells/well. After 24 h growing, the cells were incubated in fresh medium containing FITC-mPD-1 (2 μM) or premixed FITC-mPD-1 (2 μM) and Nb-Ftn (0.2 μM) for 20 min at 4°C. Cells were digested to single cells with trypsin protease (HyClone) digestion and analyzed on a flow cytometer (CytoFLEX, BECKMAN).

Primary tumor-bearing mouse model. The primary tumor-bearing mouse model was established by subcutaneously inoculating 8×10^5 B16F10 cells into the right back of C57BL/6 mice. The mice were used for further administrations or analyses when the tumor volumes reached 50 - 100 mm^3 or 100 - 150 mm^3 upon requirements.

***In vivo* NIR-II imaging.** Primary tumor-bearing mice with tumor volumes of 100 - 150 mm^3 were used for imaging. The mice were intravenously injected with PBS, ICG (2.25 mg/kg) or Nb-Ftn@ICG (equivalent to 2.25 mg/kg ICG). For *in vivo* imaging, mice were anesthetized with a Xenogen XGI-8 Gas Anesthesia apparatus (Caliper Life Science, Hopkinton, MA) and imaged with the Xenogen IVIS Lumina System (Caliper Life Science, USA) at different timepoints. For *ex vivo* imaging, mice were sacrificed 24 h post-administration, and major organs and tumors were collected and imaged with the Xenogen IVIS Lumina System.

***In vivo* PTT performance** Primary tumor-bearing mice with tumor volumes of 100 - 150 mm^3 were used for PTT analyses. The mice were intravenously injected with PBS, ICG (2.25 mg/kg) or Nb-Ftn@ICG (equivalent to 2.25 mg/kg ICG) when the tumor volumes reached 100 - 150 mm^3. PTT was applied by 808 nm laser irradiation (1 W/cm^2) on tumors for 5 min, and the temperature alterations of mice were recorded with an infrared thermal imaging camera (FOTRIC 225s).

Detection of DAMPs release *in vivo.* Primary tumor-bearing mice with tumor volumes of 100 - 150 mm^3 were used for the DAMPs release assay. The mice were intravenously injected with PBS, ICG (2.25 mg/kg) or Nb-Ftn@ICG (equivalent to 2.25 mg/kg ICG). The tumors were irradiated by laser for 5 min at 12 h post injection. After another 12 h, the mice were sacrificed, and the tumors were collected. The tumor slices were blocked with goat serum for 30 min, then the sections were incubated with individual primary antibodies against HMGB1 or CRT for 1 h at 37°C, followed by incubation with dye-conjugated secondary antibodies for 30 min at 37°C. After staining with DAPI for another 5 min, the slices were washed twice with PBS, and the images were recorded under a confocal laser scanning microscope (Vs200, Olympus).

***In vivo* therapeutic efficacy against primary tumors.** Primary tumor-bearing mice with tumor volumes of 50 - 100 mm^3 were used for the *in vivo* anti-tumor assay. The mice were randomly divided into six groups (n = 5) and intravenously administered with PBS, PBS + L, ICG (2.25 mg/kg), ICG + L (2.25 mg/kg), Nb-Ftn@ICG (equivalent to 2.25 mg/kg ICG), Nb-Ftn@ICG + L (equivalent to 2.25 mg/kg ICG). At 12 h post-injection, the mice in the "+ L" group were irradiated by an 808 nm laser (1.0 W/cm^2) for 5 min. The mice were administrated with different formulations only once, and the tumor volumes and body weights were measured every two days. The tumor volume was calculated according to the equation $V = 0.5 \times W^2 \times L$, wherein W and L represent the maximum length (mm) and minimal length (mm) of tumors, respectively. 12 days after treatment, the mice were sacrificed, and the tumors and major organs were collected for further analyses. The slices of major organs and tumors in all groups were analyzed with hematoxylin and eosin (H&E), TdT-mediated dUTP Nick-End Labeling (TUNEL) and Ki67 immunohistochemistry assays.

Detection of PD-L1 levels *in vivo.* Primary tumor-bearing mice with tumor volumes of 100 - 150 mm^3 were used for the *in vivo* anti-tumor assay. The mice were intravenously injected with Nb-Ftn (10.0 mg/kg, 0.27 nmol per 20 g mouse). At different time points (12, 24, 36, 48 h) post administration, the mice were sacrificed, and the tumors were collected. The PD-L1 levels in tumors were analyzed with Western blotting and immunohistochemistry.

***In vivo* immune response and suppression of abscopal tumors.** For the establishment of a primary and abscopal tumor-bearing mouse model, C57BL/6 mice were subcutaneously injected with 8×10^5 B16F10 cells in the left back (primary

tumor), and four days later, they were injected with 5 × 10^5 B16F10 cells in the right back (abscopal tumor). After another four days, the mice were randomly divided into seven groups (n = 5) for different treatments: PBS, (I) Nb-Ftn@ICG (equivalent to 2.25 mg/kg of ICG), (II) Nb-Ftn@ICG + Nb-Ftn (equivalent to 2.25 mg/kg of ICG), (III) Nb-Ftn@ICG + αPD-1 (equivalent to 2.25 mg/kg of ICG), (IV) Nb-Ftn@ICG + L (equivalent to 2.25 mg/kg of ICG), (V) Nb-Ftn@ICG + L + Nb-Ftn (equivalent to 2.25 mg/kg ICG), and (VI) Nb-Ftn@ICG + L + αPD-1 (equivalent to 2.25 mg/kg ICG). At 12 h after administration, the left tumors of the mice in the "+ L" group were irradiated with an 808 nm laser (1.0 W/cm^2) for 5 min, while the rest parts of the mice were covered with aluminum foil to shield them from irradiation. The mice received additional ICB therapy on days 3, 6, and 9 with Nb-Ftn (10.0 mg/kg, group II - III) or αPD-1 (7.5 mg/kg group V - VI) by intravenous injection. The body weights and the sizes of primary and abscopal tumors were monitored every two days. The mice were sacrificed 14 days after the treatments, and the tumor tissues were collected and frozen for sectioning. CD4 and CD8 levels of tumors in all groups were analyzed with immunohistochemistry evaluation of the degree of tumor infiltration of T cells.

***In vivo* anti-metastasis.** A lung metastasis model was established by intravenous injection of 3 × 10^5 B16F10 cells into the primary tumor-bearing mice five days after inoculation of the primary tumors. Mice were randomly divided into seven groups after 0.5 days and received different treatments of PBS, (I) Nb-Ftn@ICG (equivalent to 2.25 mg/kg ICG), (II) Nb-Ftn@ICG + Nb-Ftn (equivalent to 2.25 mg/kg ICG), (III) Nb-Ftn@ICG + αPD-1 (equivalent to 2.25 mg/kg ICG), (IV) Nb-Ftn@ICG + L (equivalent to 2.25 mg/kg ICG), (V) Nb-Ftn@ICG + L + Nb-Ftn (equivalent to 2.25 mg/kg ICG), and (VI) Nb-Ftn@ICG + L + αPD-1 (equivalent to 2.25 mg/kg ICG), the same dosage as described above for the anti-abscopal tumor assay. On day 14, all mice were sacrificed, and the lungs were excised and analyzed with photographic imaging and H&E staining assays.

Ethics statement. Studies involving the use of animals were completed under the guidelines of the National Regulation of China for Care and Use of Laboratory Animals, and the protocol was approved by the University of Science and Technology of China Animal Experiment Center Committee with the assigned accreditation number PXHG-LYZ2021041519.

References

[30] D. Y. Lin, Y. Tanaka, M. Iwasaki, A. G. Gittis, H. P. Su, B. Mikami, T. Okazaki, T. Honjo, N. Minato, D. N. Garboczi, *Proc Natl Acad Sci U S A* **2008**, *105*, 3011-3016.

[31] T. Wu, H. Huang, Y. Sheng, H. Shi, Y. Min, Y. Liu, *J Mater Chem B* **2018**, *6*, 1011-1017.

[32] K. Broos, M. Keyaerts, Q. Lecocq, D. Renmans, T. Nguyen, D. Escors, A. Liston, G. Raes, K. Breckpot, N. Devoogdt, *Oncotarget* **2017**, *8*, 41932-41946.

2. Supplemental Figures

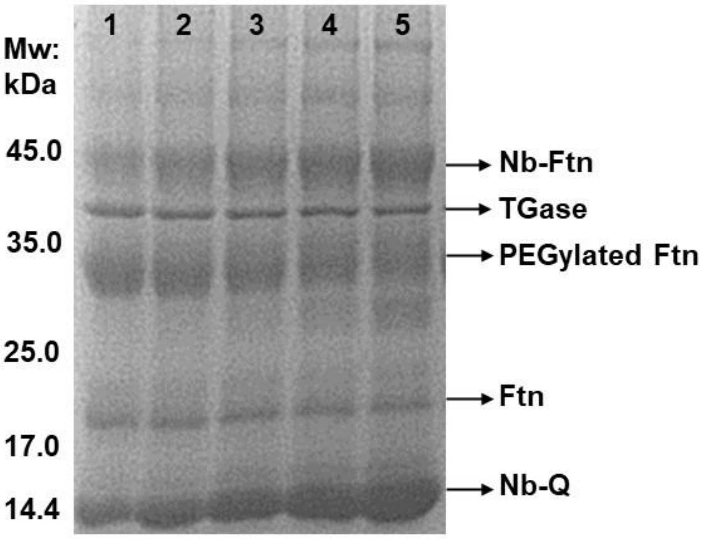

Figure S1. SDS-PAGE analyses of the conjugation of Nb-Q with PEGylated Ftn@ICG via TGase-catalyzed ligation. The molar ratios of Nb-Q to PEGylated Ftn@ICG in lanes 1-5 are 5:1 (lane 1), 10:1 (lane 2), 15:1 (lane 3), 20:1 (lane 4), and 30:1 (lane 5), respectively. 1 mg/mL of TGase was used in all reactions in this work. A molar ratio of 20:1 of Nb-Q to PEGylated Ftn (lane 4) was used for the conjugations in this work.

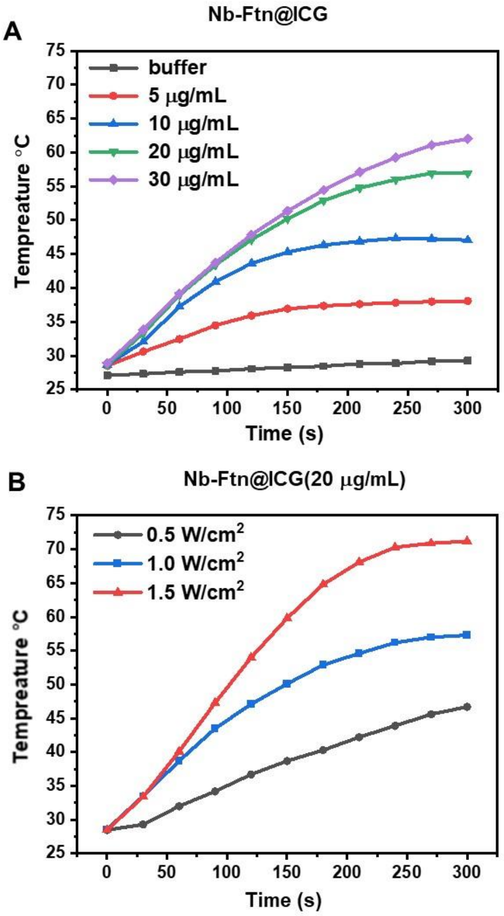

Figure S2. Photothermal effect of Nb-Ftn@ICG in solution 808 nm laser irradiation. (A) Nb-Ftn@ICG at different concentrations (equivalent to 0 - 30 μg/mL free ICG) with laser irradiation at 1 W/cm^2 for 5 min. (B) Laser irradiation to Nb-Ftn@ICG (equivalent to 20 μg/mL free ICG) for 5 min in different powers.

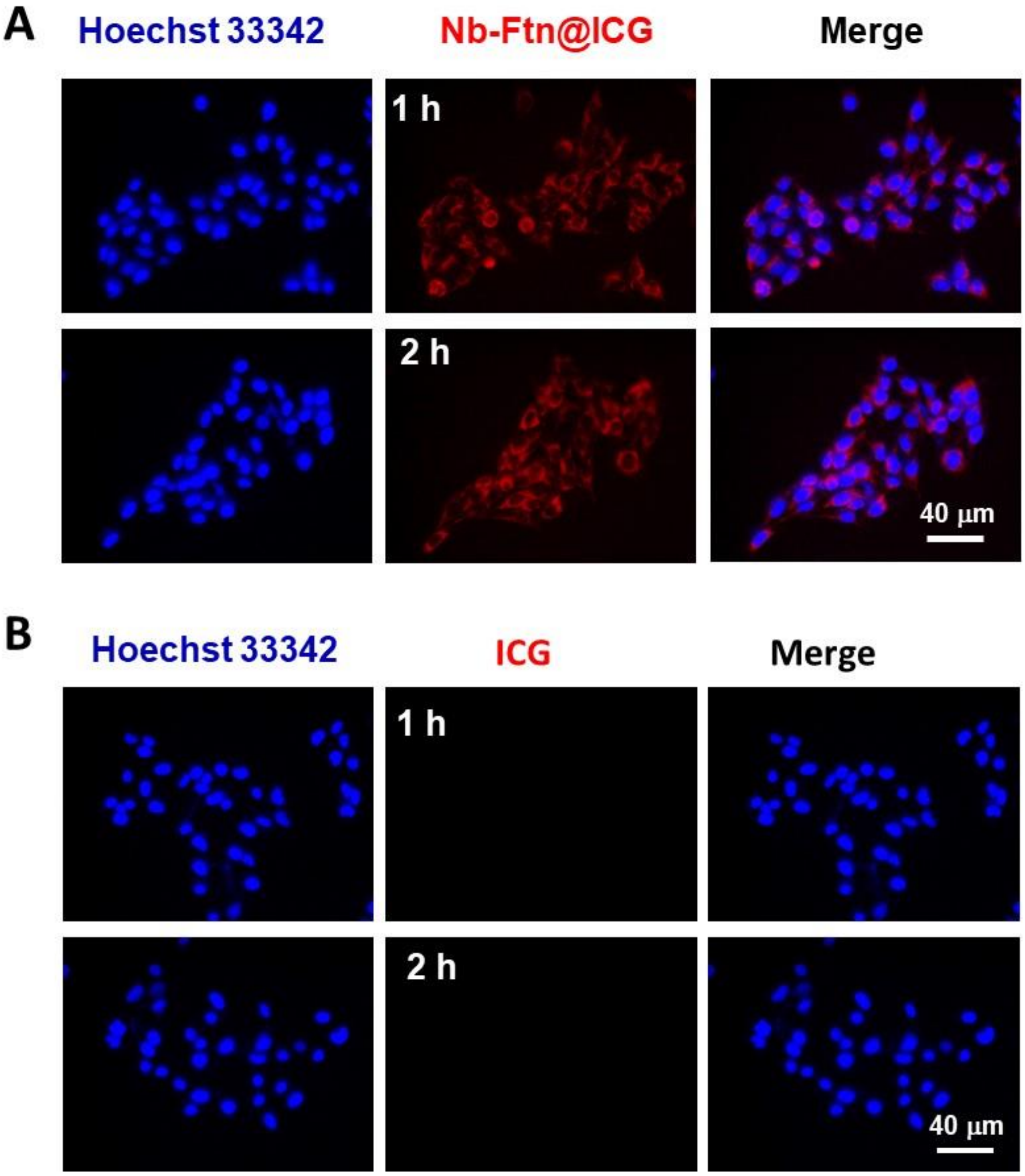

Figure S3. Cellular uptake of Nb-Ftn@ICG (A) or free ICG (B) measured with fluorescence imaging. Images were recorded after incubation of cells with Nb-Ftn@ICG (equivalent to 0.6 μM ICG) or ICG (0.6 μM) for 1 or 2 h. ICG gave red fluorescence, and nuclei were stained by Hoechst 33342 (blue). Scale bar = 40 μm.

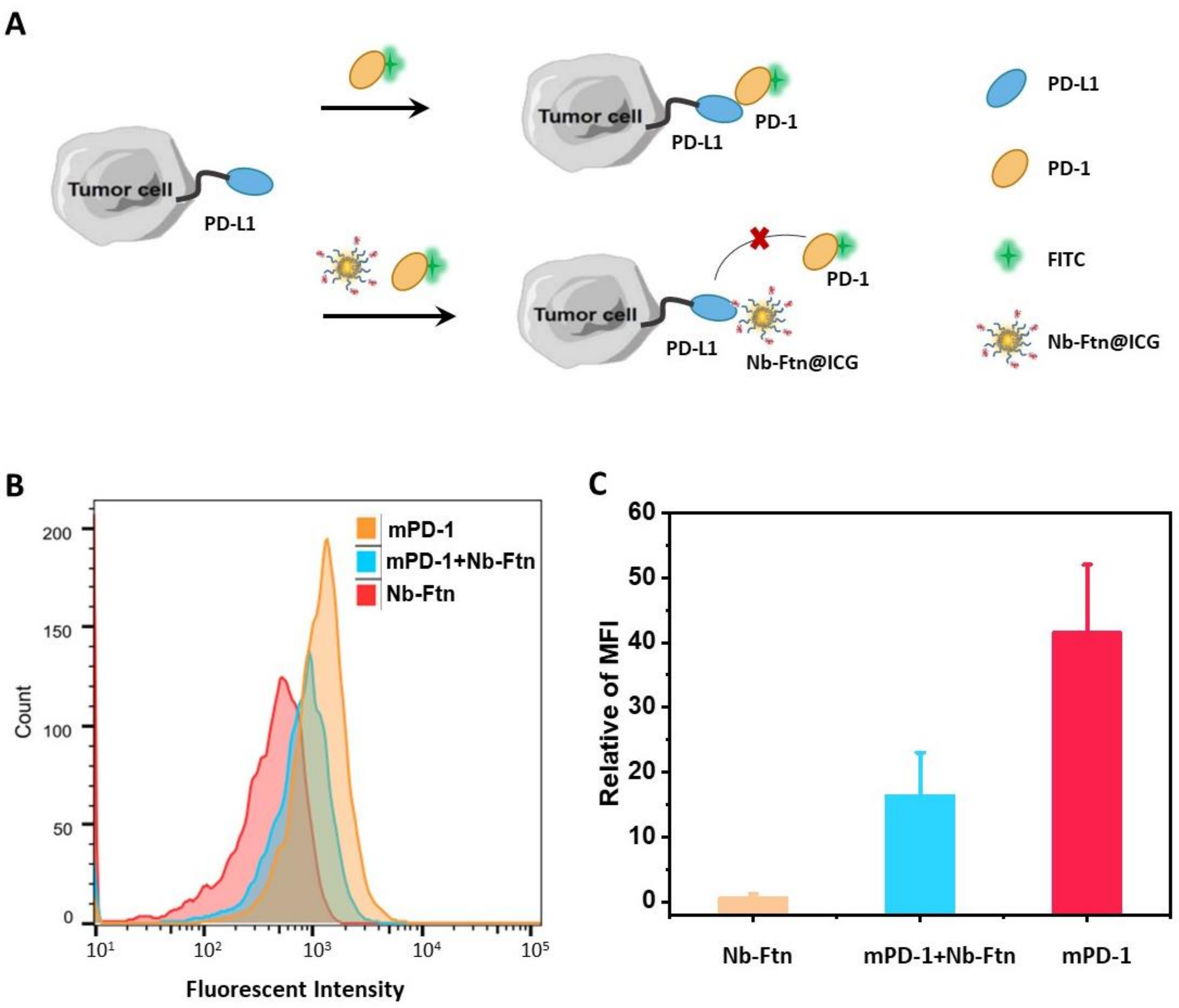

Figure S4. Flow cytometry analysis of blockade of the PD-1/PD-L1 interaction. (A) Schematic illustration of the blockade of the PD-1/PD-L1 interaction on the cell surface by Nb-Ftn. mPD-1 (recombinant extracellular domain of the murine PD-1) was labeled with the green dye FITC (green), and Nb-Ftn encapsulated ICG. (B) Flow cytometry analysis of B16F10 cells treated with mPD-1 (2 μM), Nb-Ftn@ICG (0.2 μM), or their mixture for 20 min. mPD-1 was labeled by FITC (green), and Nb-Ftn encapsulated ICG. The wavelength was set to 488 nm for FITC excitation. (C) Quantification of the relative mean fluorescent intensity (MFI) values of the flow cytometry measurement. Error bars represent three parallel experiments.

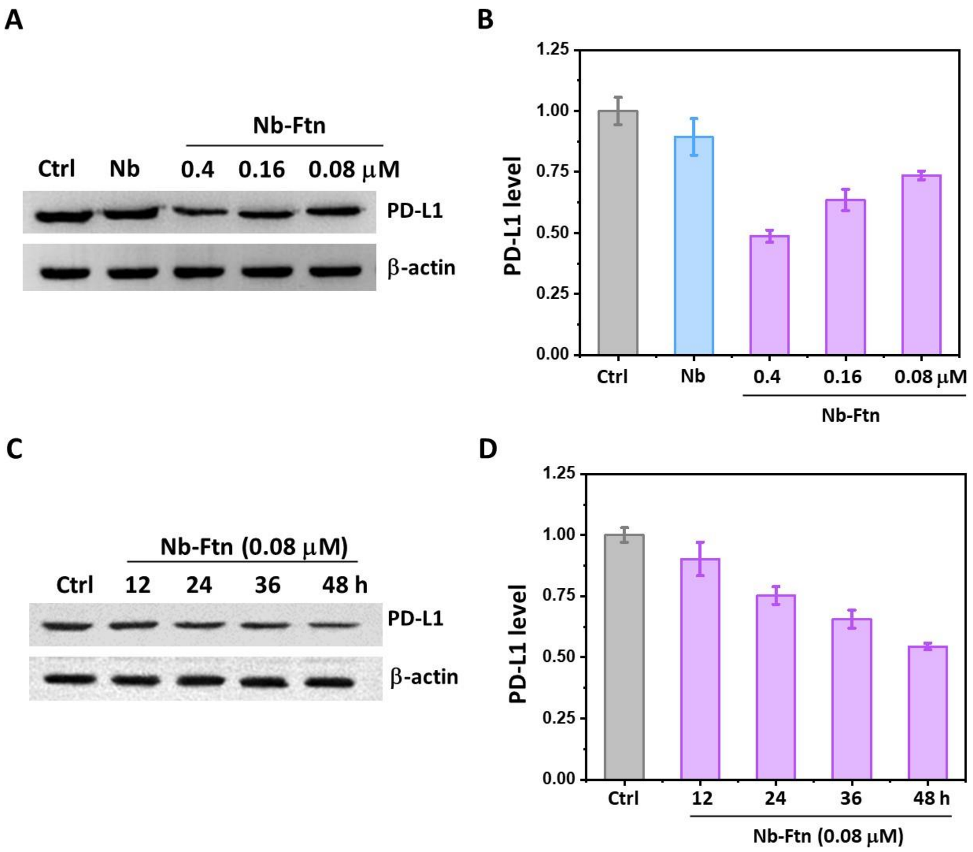

Figure S5. The cellular PD-L1 level downregulated by Nb-Ftn. The PD-L1 level changes were obtained by measuring the change PD-L1/β-actin ratios on Western blotting analysis. Cells were treated with different concentrations of Nb-Ftn for 24 h (A, B) and or 0.08 μM Nb-Ftn for different time (C, D). The band quantification was processed by software Image J (B, D).

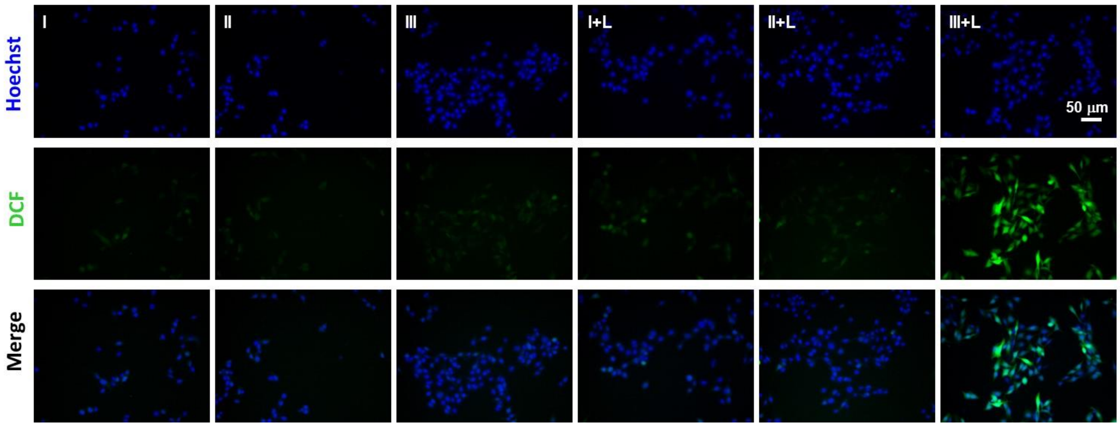

Figure S6. Fluorescence imaging of ROS generation in cells. Cells were treated with PBS (I), ICG (II) or Nb-Ftn@ICG (III) at 37°C for 2 h. After changing the medium to fresh medium, 808 nm laser irradiation (1 W/cm^2 for 5 min) was applied to the cells labeled with "+ L". Then, the cells were incubated with the ROS probe DCFH-DA and Hoechst before CLSM imaging. Scale bar = 50 μm.

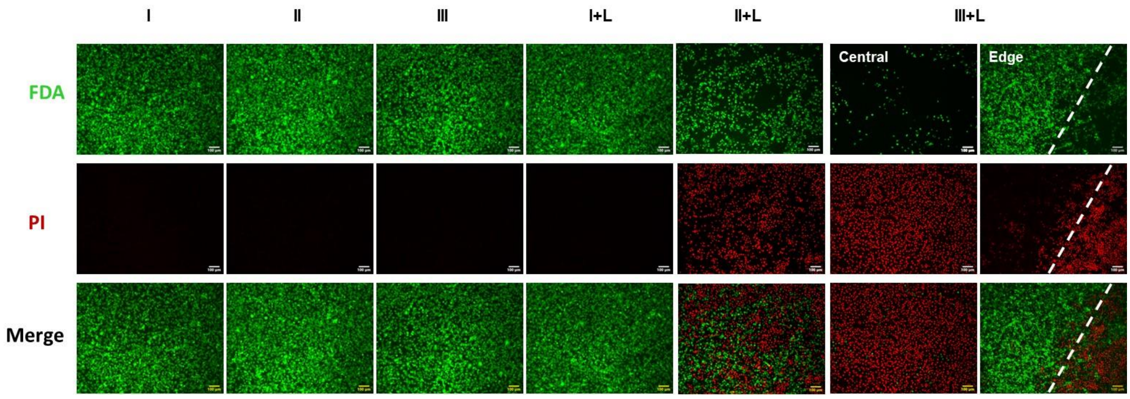

Figure S7. Fluorescent imaging of live/dead cell assay. Live cells were stained with FDA (green), and dead cells were stained with PI (red). Cells were treated with PBS (I), ICG (II, 12 μg/mL) or Nb-Ftn@ICG (III, equivalent to 12 μg/mL of free ICG). "+ L" denotes the cells with laser irradiation. Cells treated with Nb-Ftn@ICG are shown in the center of the laser spot (annotated with "Central") and at the edge of the laser spot (annotated with "Edge"). Scale bar = 100 μm.

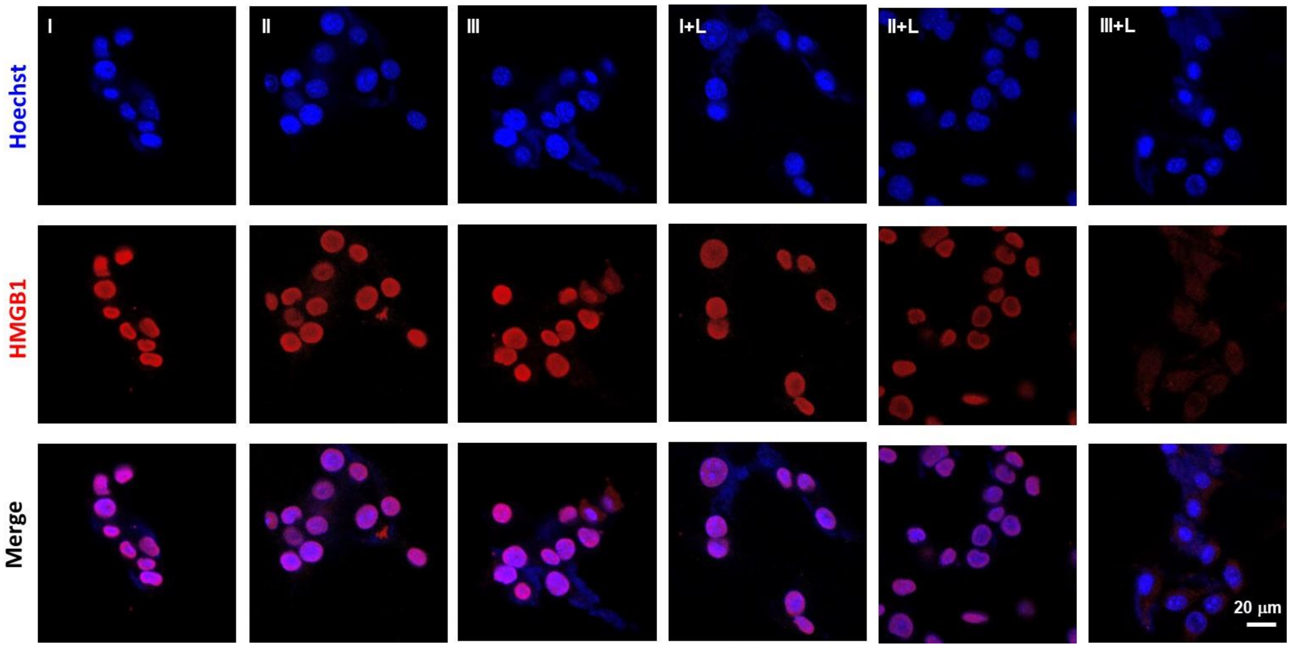

Figure S8. HMGB1 release measured with CLSM imaging. Cells were treated with PBS (I), ICG (II, 12 μg/mL) or Nb-Ftn@ICG (III, equivalent to 12 μg/mL of free ICG) for 2 h, followed by laser irradiation for 5 min (to the groups labeled with "+ L"). HMGB1 was labeled with anti-HMGB1 antibody (labeled with red probe), and nuclei were stained with Hoechst 33342 (blue). Scale bar = 20 μm.

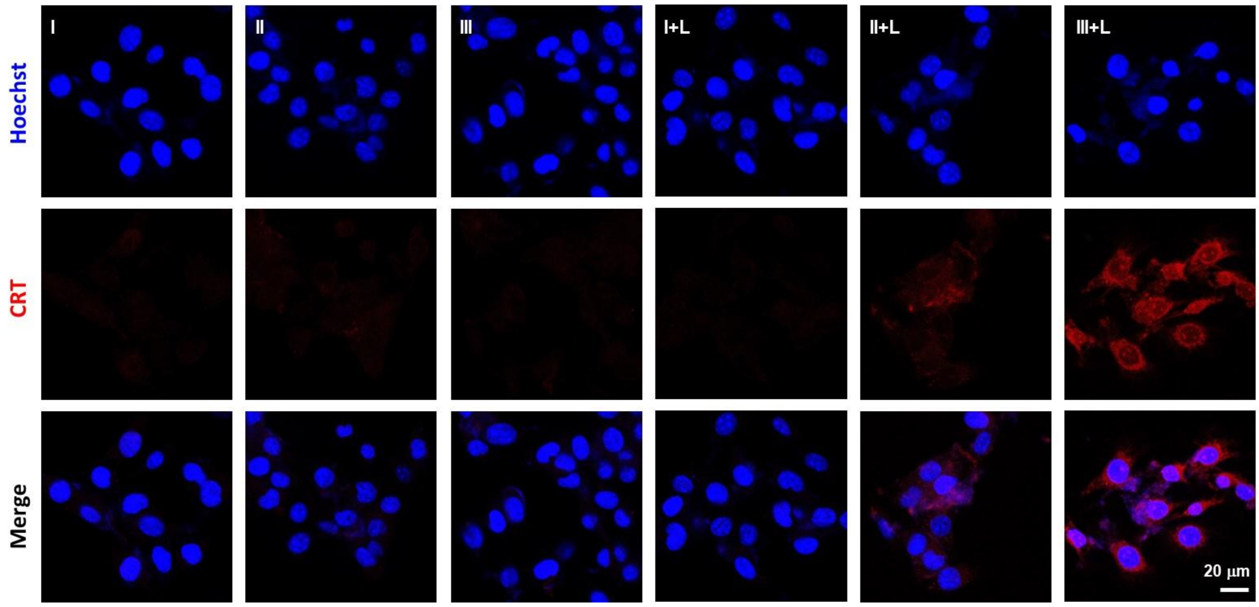

Figure S9. CRT exposure measured with CLSM imaging. Cells were treated with PBS (I), ICG (II, 12 μg/mL) or Nb-Ftn@ICG (III, equivalent to 12 μg/mL free ICG) for 2 h, followed by laser irradiation for 5 min (to the groups labeled with "+ L"). CRT was labeled with anti-CRT antibody (followed by a further incubation with secondary antibody labeled with red probe), and nuclei were stained with Hoechst 33342 (blue). Scale bar = 20 μm.

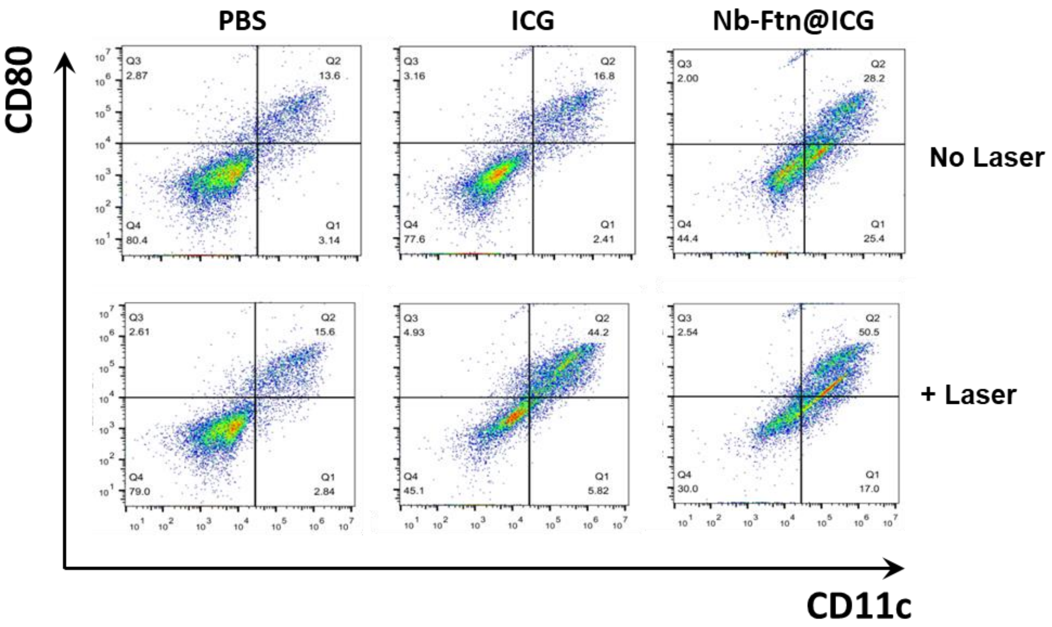

Figure S10. Flow cytometry analysis of DC maturation. DC cells were incubated with B16F10 cells pretreated with PBS (I), ICG (II), or Nb-Ftn@ICG (III). Laser irradiation (808 nm, 1 W/cm^2) was applied for 5 min.

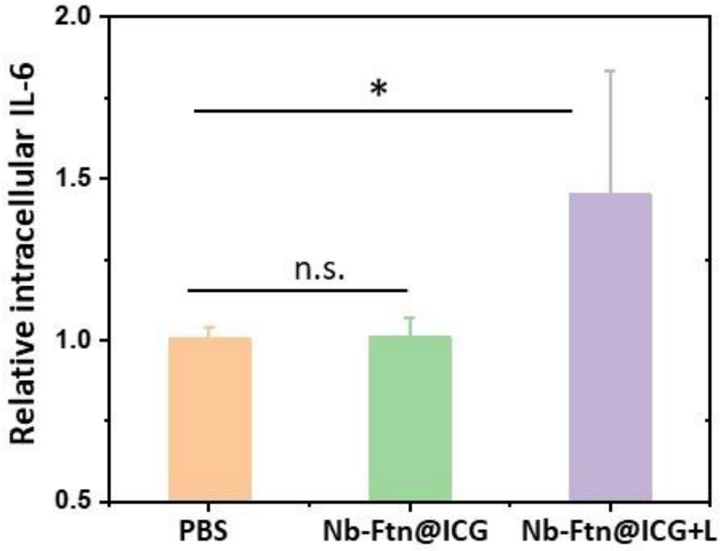

Figure S11. Relative cytokine levels of IL-6 in DC suspensions were detected by ELISA kits (n = 3, *P < 0.05). B16F10 cells were treated with PBS, Nb-Ftn@ICG, or Nb-Ftn@ICG + L. "L" stands for laser irradiation (808 nm, 1 W/cm^2) for 5 min. 4 h after of laser irradiation, B16F10 cells were incubated with DC cells for 24 h, then the DC suspensions were collected and detected by ELISA kits.

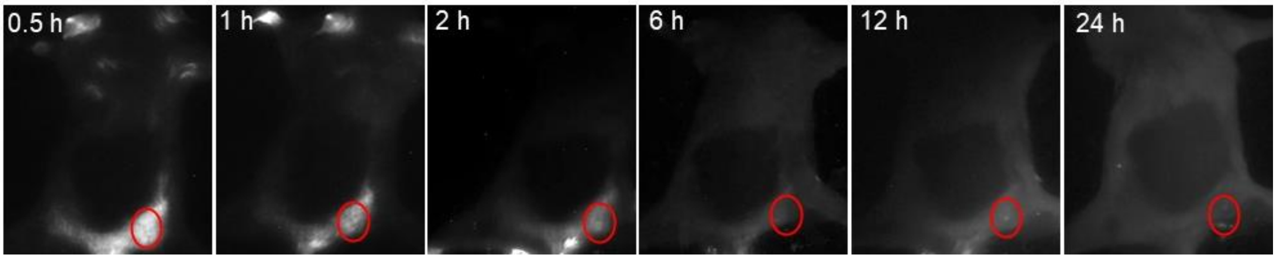

Figure S12. *In vivo* NIR-II imaging of B16F10 tumor-bearing mice. ICG (2.25 mg/kg) was intravenously injected, and images were recorded at different time points after injection. The red circles mark the location of tumors.

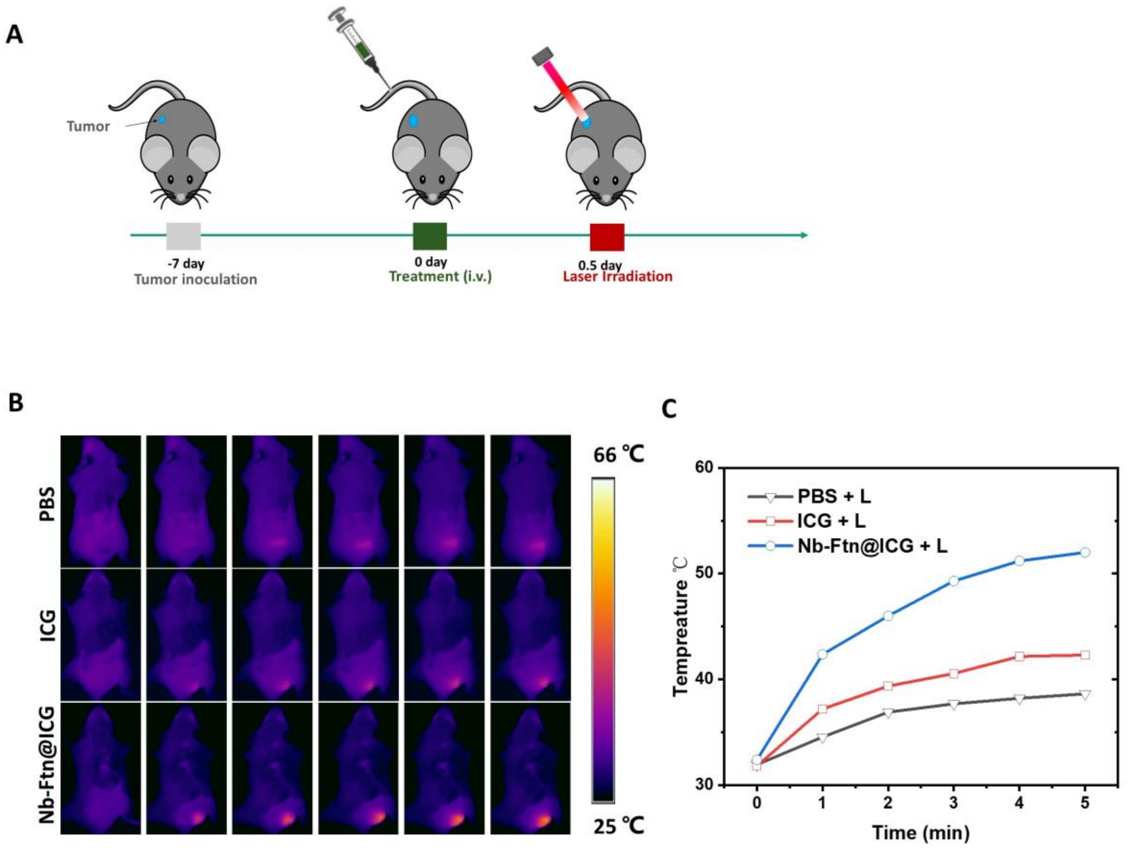

Figure S13. *In vivo* photothermal effect of Nb-Ftn@ICG. (A) Tumor bearing mice were treated with ICG (2.25 mg/kg) or Nb-Ftn@ICG (equivalent to 2.25 mg/kg ICG) via intravenous injection. 0.5 day after administration, tumors of mice were irradiated with an 808 nm laser (1.0 W / cm^2) for 5 min. (B) Photothermal images of tumors of mice at different time after laser irradiation. (C) Profile of temperature change of tumors after laser irradiation for 5 time.

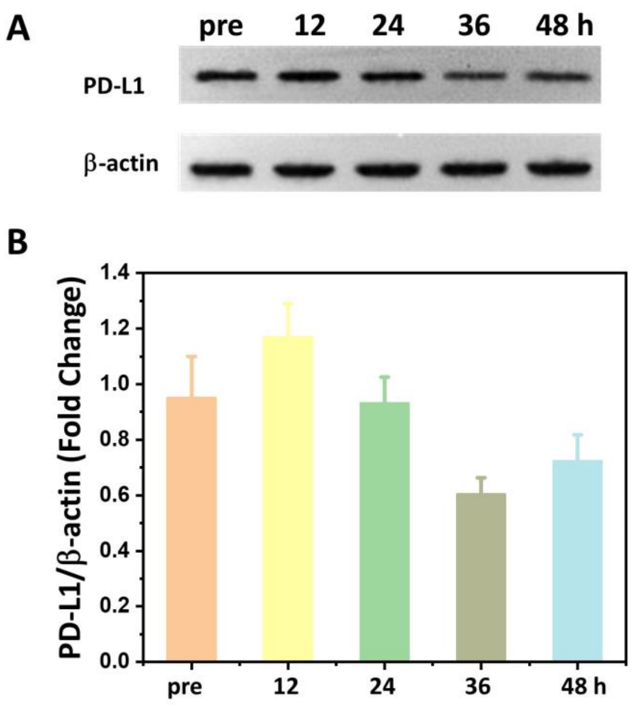

Figure S14. The total PD-L1 levels (A) and the quantification (B) in tumors excised from mice at different times after a single injection of Nb-Ftn (10.0 mg/kg, 0.27 nmol per 20 g mouse). The bands were quantified by software Image J.

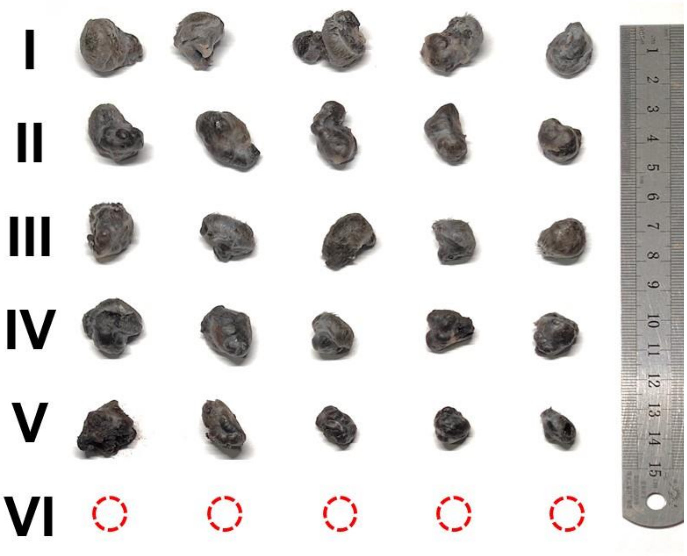

Figure S15. Photographs of excised tumors from mice after different treatments of PBS (I), ICG (II), Nb-Ftn@ICG (III), PBS + L (IV), ICG + L (V) or Nb-Ftn@ICG + L (VI). “+ L” indicates the mice that received laser irradiation (*n* = 5). Red circles indicate that the tumor was completely ablated.

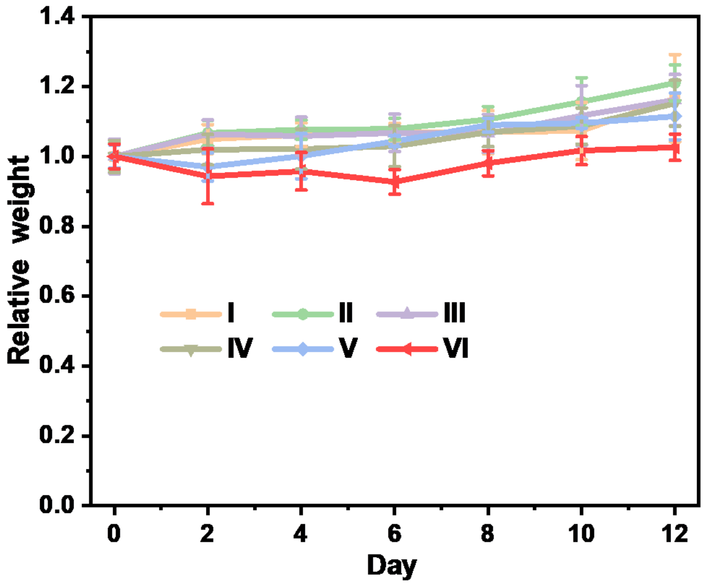

Figure S16. Weights of B16F10 tumor-bearing mice during the treatments of PBS (I), ICG (II), Nb-Ftn@ICG (III), PBS + L (IV), ICG + L (V) or Nb-Ftn@ICG + L (VI). "+ L" indicates the mice that received laser irradiation.

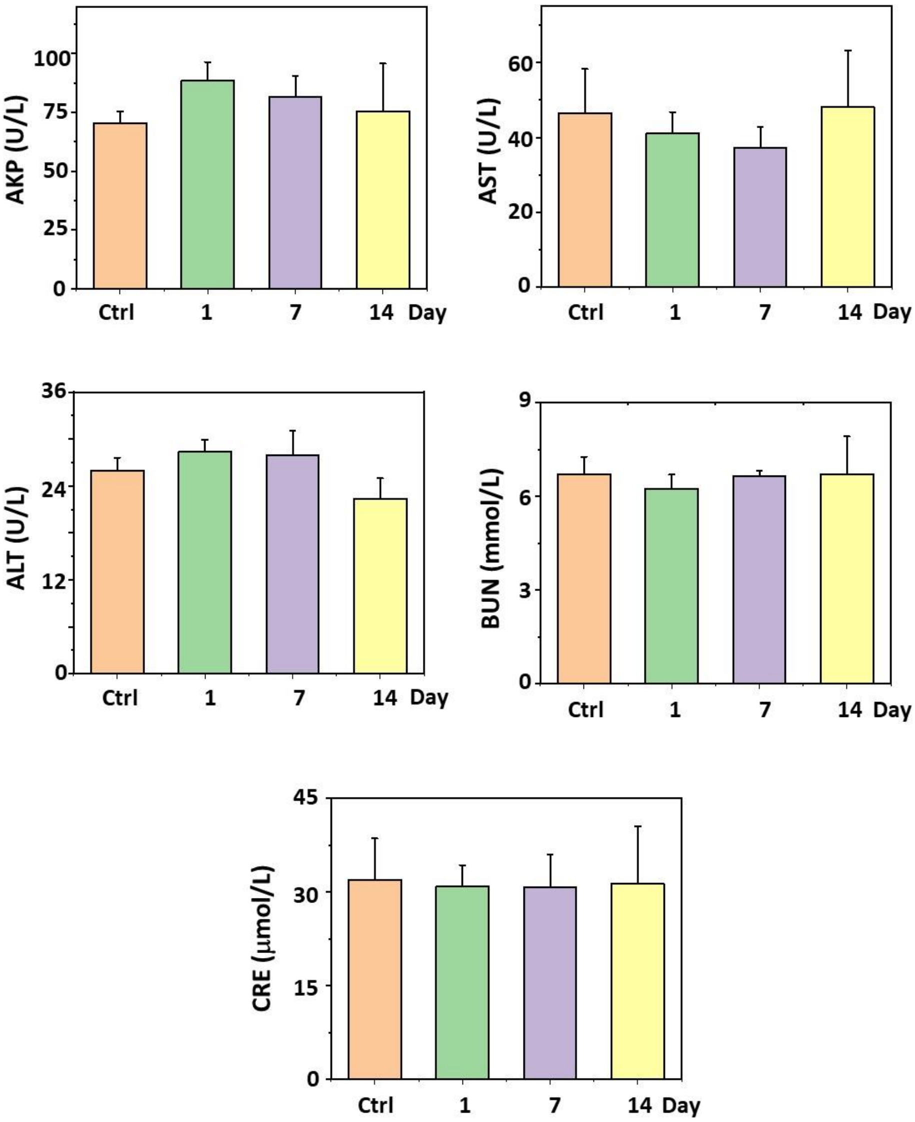

Figure S17. Blood biochemical tests of healthy mice after Nb-Ftn@ICG treatment. Nb-Ftn@ICG (equivalent to 2.25 mg/kg of free ICG) was intravenously injected, and the blood biochemical parameters in serum were measured at different time, including the levels of alkaline phosphatase (AKP), aspartate aminotransferase (AST), alanine aminotransferase (ALT), blood urea nitrogen (BUN), and creatinine (CRE).

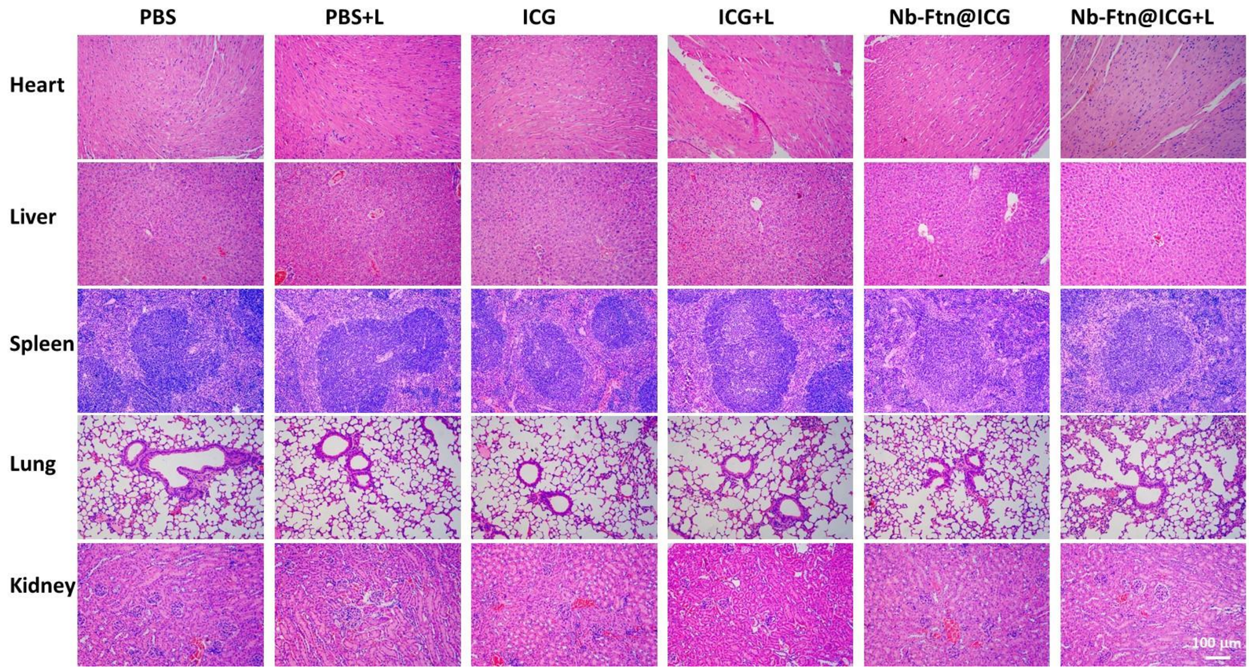

Figure S18. Representative H&E staining images of major organs of B16F10-bearing mice after different treatments. Scale bar = 100 μm.

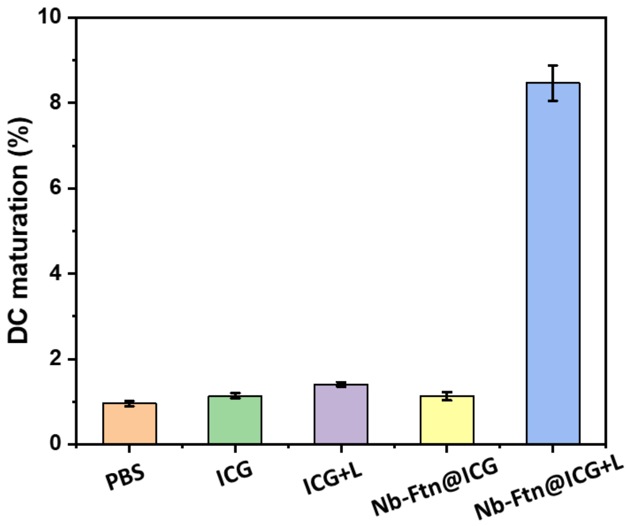

Figure S19. Flow cytometry analysis of the percentage of mature DCs ($CD86^+/CD80^+$) in tumors on day 3 after treatment. "+ L" denotes the group that received laser irradiation.

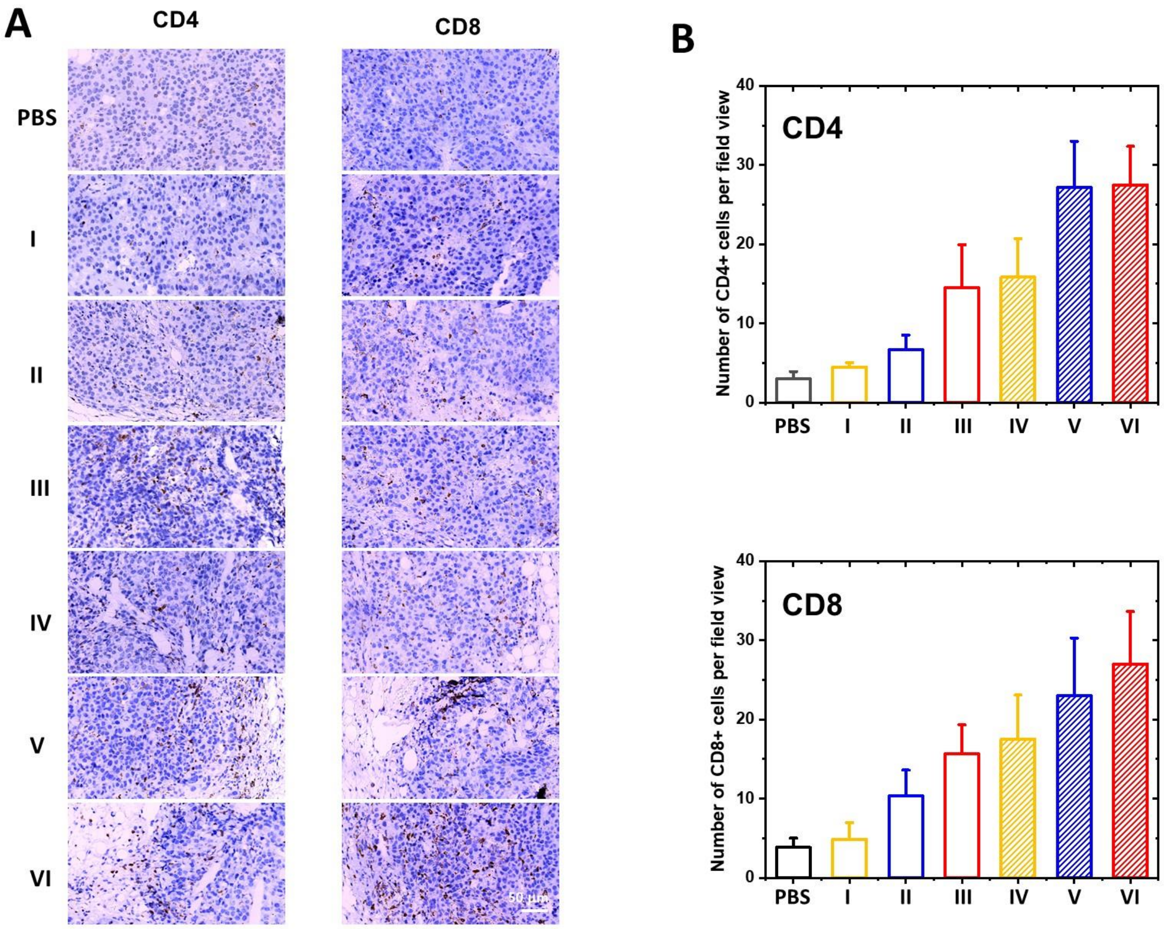

Figure S20. (A) Immunohistochemistry analysis of distal tumors excised from mice after treatment of Nb-Ftn@ICG (I), Nb-Ftn@ICG + Nb-Ftn (II), Nb-Ftn@ICG + αPD-1 (III), Nb-Ftn@ICG + L (IV), Nb-Ftn@ICG + L + Nb-Ftn (V), or Nb-Ftn@ICG + L + αPD-1 (VI). "+ L" denotes laser irradiation (*n* = 5), wherein $CD4^+$ and $CD8^+$ T cells were stained. Scale bar = 50 μm. (B) Quantification of the number of $CD4^+$ or $CD8^+$ T cells in 6 view of immunohistochemistry analysis result (6 views similar to A).

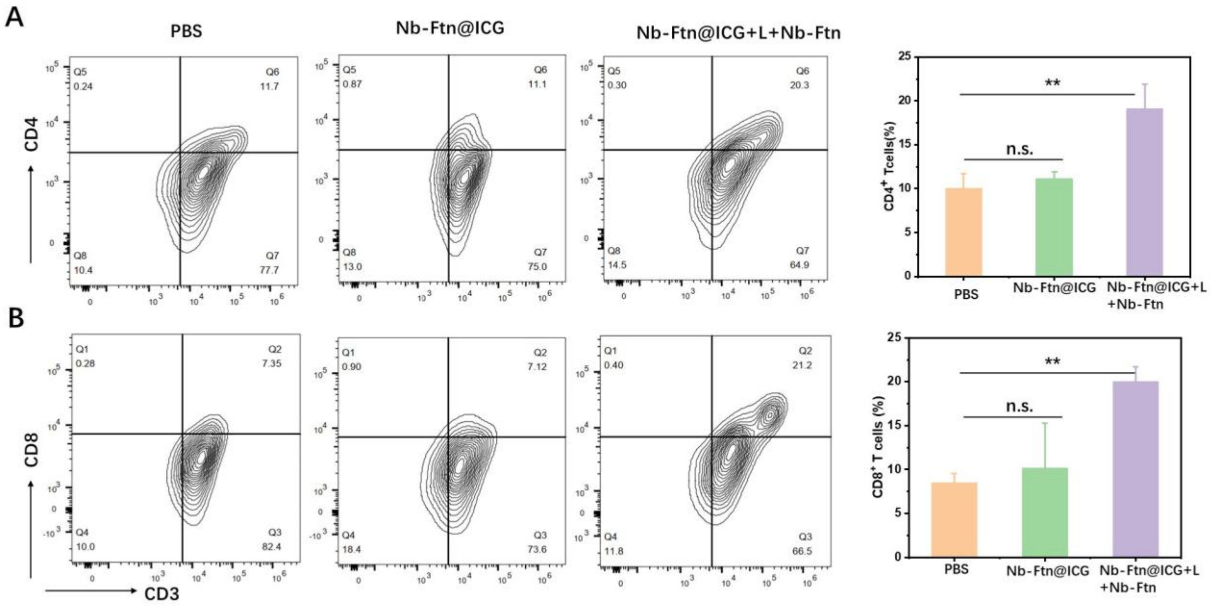

Figure S21. Representative flow cytometry plots of T cells in distal tumors 5 days post various treatments (n =3, *P < 0.05,**P < 0.01). Mice with bilateral B16F10 tumors were divided into 3 groups and treated with PBS, Nb-Ftn@ICG, or Nb-Ftn@ICG + L + Nb-Ftn. The mice in the Nb-Ftn@ICG + L + Nb-Ftn group were treated with Nb-Ftn@ICG and received laser irradiation 0.5 days post injection, followed by Nb-Ftn treatment after 3 days. The numbers of $CD3^+$, $CD4^+$ and $CD8^+$ T cells in distal tumors were measured by flow cytometry.

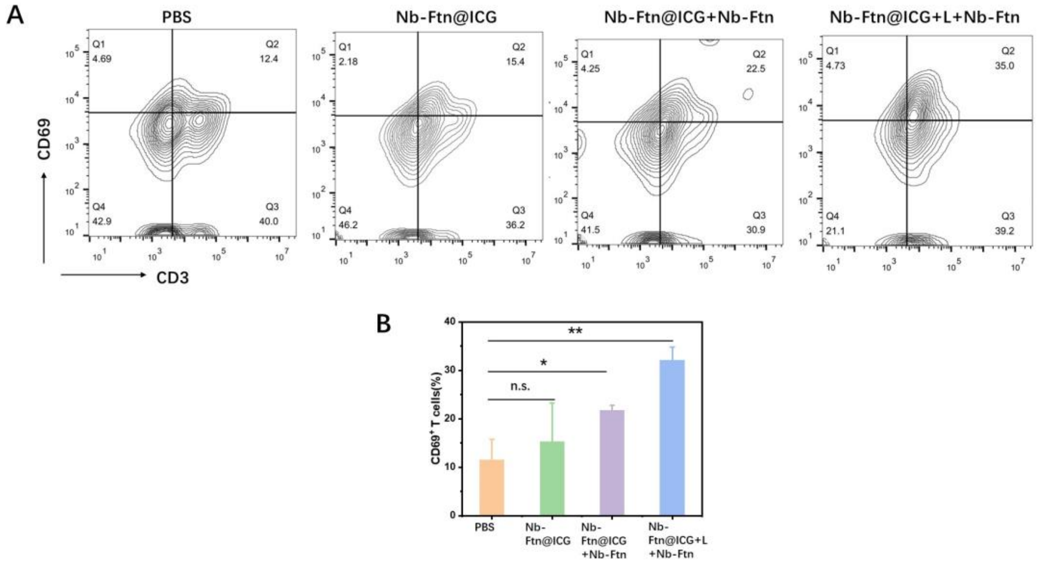

Figure S22. (A) Representative flow cytometry plots of active T cells in distal tumors 8 days post various treatments (n =3, *P < 0.05,**P < 0.01). (B) Proportion of CD69$^+$ T cells after the treatments. Mice with bilateral B16F10 tumors were divided into 4 groups and treated with PBS, Nb-Ftn@ICG, Nb-Ftn@ICG + Nb-Ftn, or Nb-Ftn@ICG + L + Nb-Ftn. The mice in the Nb-Ftn@ICG + L + Nb-Ftn group were treated with Nb-Ftn@ICG and received laser irradiation 0.5 days post injection, followed by Nb-Ftn treatment on the day 3 and day 6. The mice in the Nb-Ftn@ICG + Nb-Ftn group were received the same treatment as Nb-Ftn@ICG + L + Nb-Ftn group except for laser irradiation. The numbers of CD3$^+$ and CD69$^+$ T cells in distal tumors were measured by flow cytometry.

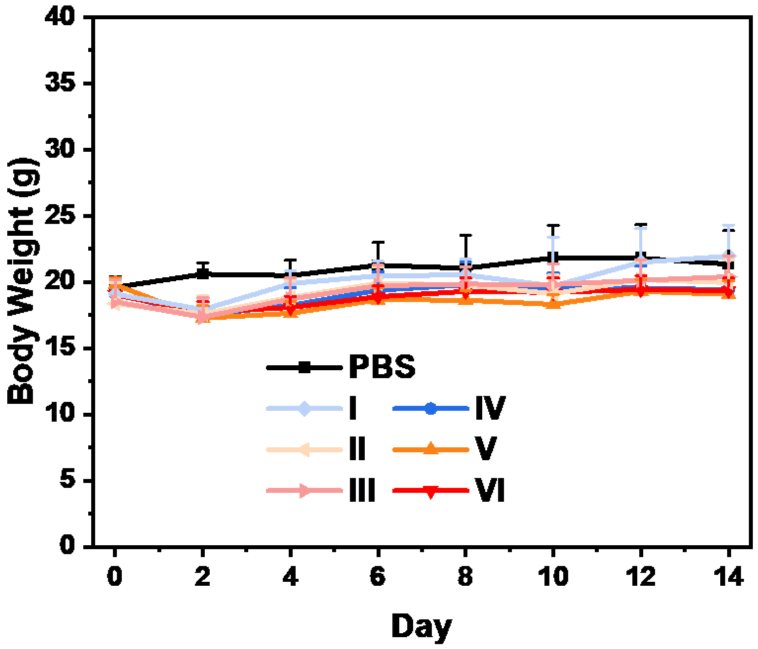

Figure S23. Weights of B16F10 tumor-bearing mice during the treatment. The mice received treatment of PBS, Nb-Ftn@ICG (I), Nb-Ftn@ICG + Nb-Ftn (II), Nb-Ftn@ICG + αPD-1 (III), Nb-Ftn@ICG + L (IV), Nb-Ftn@ICG + L + Nb-Ftn (V), or Nb-Ftn@ICG + L + αPD-1 (VI). “+ L” denotes the group that received laser irradiation.

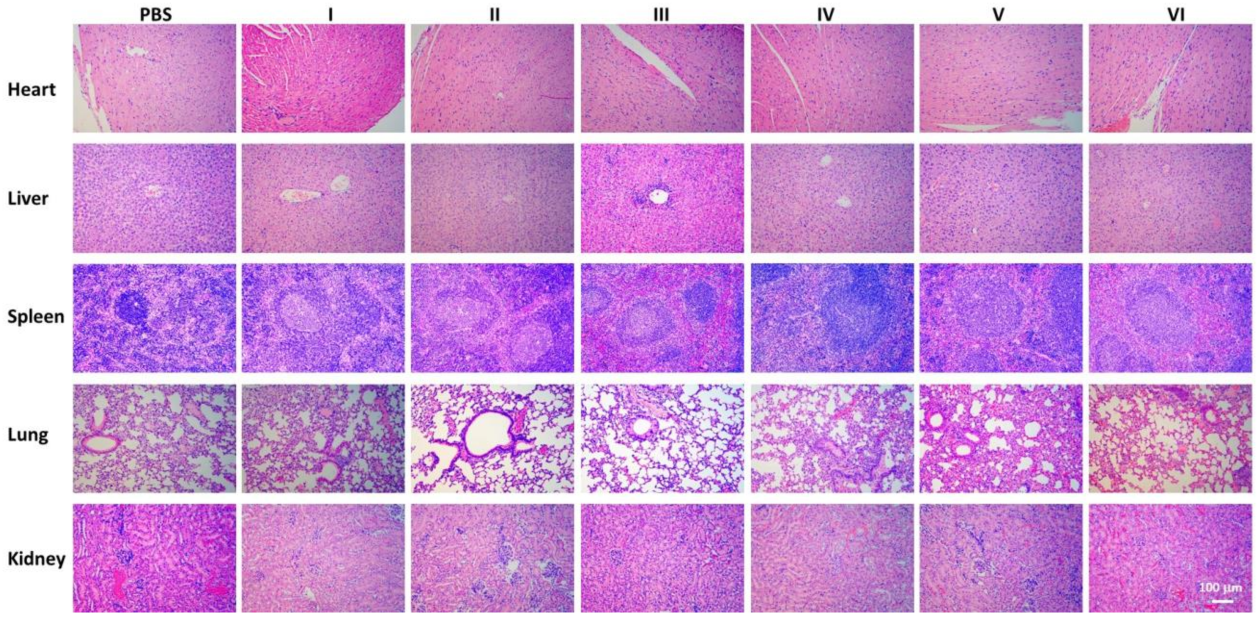

Figure S24. Representative H&E staining images of major organs of bilateral B16F10-bearing mice. The mice received the treatment of Nb-Ftn@ICG (I), Nb-Ftn@ICG + Nb-Ftn (II), Nb-Ftn@ICG + αPD-1 (III), Nb-Ftn@ICG + L (IV), Nb-Ftn@ICG + L + Nb-Ftn (V), or Nb-Ftn@ICG + L + αPD-1 (VI). "+ L" denotes the group that received laser irradiation. Scale bar = 100 μm.

3. Full Images of Western Blotting

The full images of upper portion in Figure 2D:

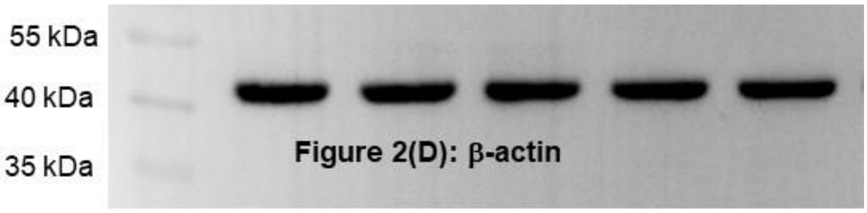

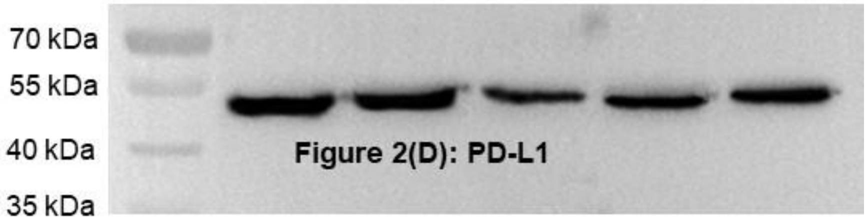

The full images of lower portion in Figure 2D:

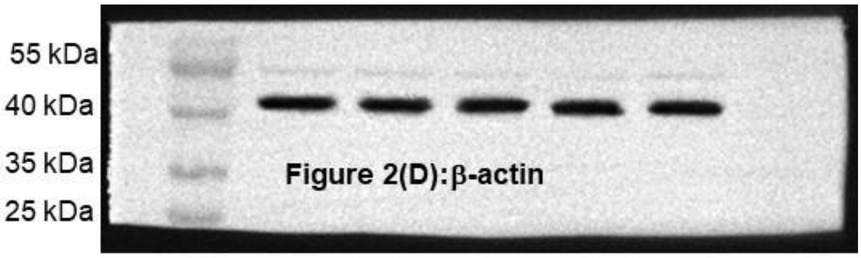

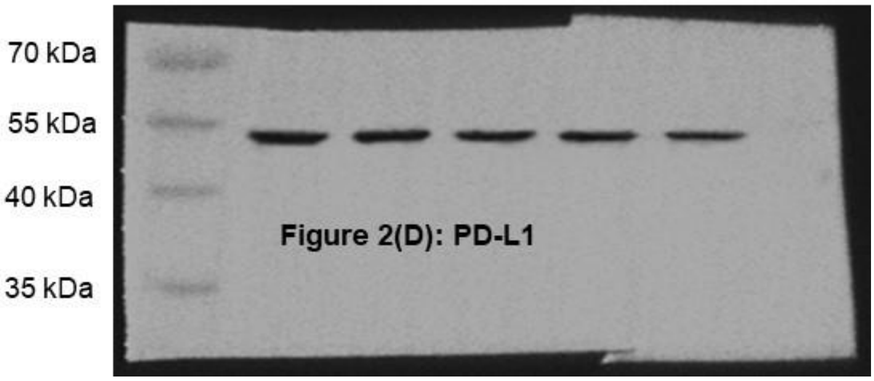

The full images of Figure 4C:

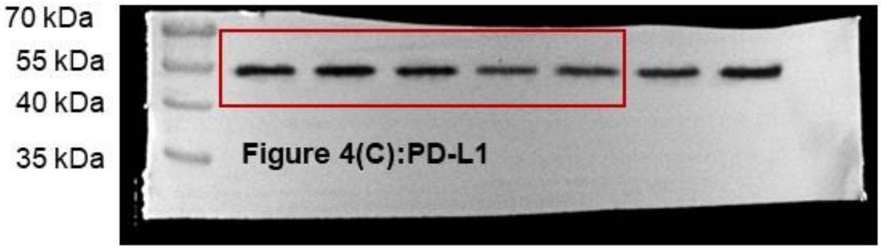

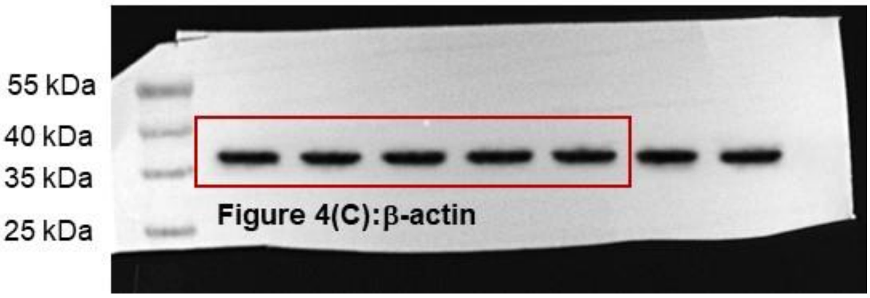

Supplement: Supplementary file 1 — Supporting Information [file ADVS-11-2308248-s001.pdf]
